# Comparative Analysis of Protocols to Induce Human CD4+Foxp3+ Regulatory T Cells by Combinations of IL-2, TGF-beta, Retinoic Acid, Rapamycin and Butyrate

**DOI:** 10.1371/journal.pone.0148474

**Published:** 2016-02-17

**Authors:** Angelika Schmidt, Matilda Eriksson, Ming-Mei Shang, Heiko Weyd, Jesper Tegnér

**Affiliations:** 1 Unit of Computational Medicine, Center for Molecular Medicine, Department of Medicine, Karolinska Institutet & Karolinska University Hospital, Stockholm, Sweden; 2 Division of Immunogenetics, Tumor Immunology Program, German Cancer Research Center (DKFZ), Heidelberg, Germany; University of Lisbon, PORTUGAL

## Abstract

Regulatory T cells (Tregs) suppress other immune cells and are critical mediators of peripheral tolerance. Therapeutic manipulation of Tregs is subject to numerous clinical investigations including trials for adoptive Treg transfer. Since the number of naturally occurring Tregs (nTregs) is minute, it is highly desirable to develop a complementary approach of inducing Tregs (iTregs) from naïve T cells. Mouse studies exemplify the importance of peripherally induced Tregs as well as the applicability of iTreg transfer in different disease models. Yet, procedures to generate iTregs are currently controversial, particularly for human cells. Here we therefore comprehensively compare different established and define novel protocols of human iTreg generation using TGF-β in combination with other compounds. We found that human iTregs expressed several Treg signature molecules, such as Foxp3, CTLA-4 and EOS, while exhibiting low expression of the cytokines Interferon-γ, IL-10 and IL-17. Importantly, we identified a novel combination of TGF-β, retinoic acid and rapamycin as a robust protocol to induce human iTregs with superior suppressive activity *in vitro* compared to currently established induction protocols. However, iTregs generated by these protocols did not stably retain Foxp3 expression and did not suppress *in vivo* in a humanized graft-*versus*-host-disease mouse model, highlighting the need for further research to attain stable, suppressive iTregs. These results advance our understanding of the conditions enabling human iTreg generation and may have important implications for the development of adoptive transfer strategies targeting autoimmune and inflammatory diseases.

## Introduction

CD4+CD25+Foxp3+ regulatory T cells (Tregs) play an indispensable role in the immune system as they are involved in the prevention of autoimmune disease, allergies and infection-induced organ pathology by suppression of other immune cells [1]. However, Tregs can also dampen immune responses against tumors in several settings [2]. Therefore, therapeutic manipulation of Treg number and function is subject to intense clinical investigations.

Foxp3 was identified as a lineage-defining transcription factor (TF) for Tregs in mice and humans, and loss of Foxp3 leads to severe lethal autoimmune disease in mice and men [1]. Yet, Foxp3 cannot serve as a specific marker for Tregs but their suppressive function has to be determined, because human conventional CD4+CD25- T cells (Tcons) transiently express intermediate Foxp3 amounts upon activation [3,4]. Only recently, transient expression of low Foxp3 levels without commitment to the Treg lineage was also shown for murine T cells [5]. Contradictory reports leave it unclear whether Foxp3 expression is sufficient to confer suppressive abilities, whereas necessity of Foxp3 for Treg function is undisputed: Foxp3, in conjunction with several other TFs, activates or represses the expression of Treg signature genes [6,7]. Significant progress has been made in elucidating the regulation of Foxp3 expression. Activation of the Foxp3 gene is achieved by binding of several TFs to its promoter and intronic Conserved Non-coding DNA Sequences (CNSs) [8,9]. These TFs are activated *via* T cell receptor (TCR), IL-2 and TGF-β signaling and, depending on which CNS they act on, are implicated in either Foxp3 induction (CNS3), maintenance (CNS2) or TGF-β-enhanced expression (CNS1). It seems that a fine balance of TCR signal intensity, timing and quality defines the optimal conditions allowing for Foxp3 induction, and furthermore, TGF-β can decrease the sensitivity towards too strong TCR stimulation [10–12]. Foxp3 expression is negatively regulated by inactivation of the Foxp3-inducing TFs Foxo1 and Foxo3a through the Akt/mTOR pathway, which is activated largely by CD28 costimulatory signals, but also IL-2R and TCR signaling cross-talk with Akt *via* the kinase PI3K [10,13,14]. Along these lines, strong costimulation was suggested to inhibit Treg induction [10,15–17]. Hence, the clinically approved mTOR inhibitor rapamycin (Rapa) promotes Foxp3 expression as shown for murine Tregs [18–21]. Also for human Tregs, Rapa has been successfully used in expansion of Tregs, while at the same time it prevents growth of Tcons [22,23]. An additional layer of complexity is added by DNA methylation and histone modifications at the Foxp3 locus and, interestingly, an epigenetic “Treg signature” can be established independently of Foxp3 [24,25]. In particular, the CNS2 comprises the so-called Treg-specific demethylated region (TSDR), which includes several CpG motifs, demethylation of which is crucial for stable maintenance of Foxp3 expression: The TSDR is demethylated exclusively in stable Tregs while it is methylated in naïve and activated Tcons as well as in ex-Tregs that have lost Foxp3 [26–28].

Peripheral tolerance is ensured not only by thymus-derived Tregs (tTregs, often called nTregs) but also involves various populations of peripherally induced Tregs (pTregs) [29–31] of which we will here confine to Foxp3+ Tregs only. tTregs are thought to be most important in maintaining tolerance to self-antigens, while pTregs are supposed to ensure tolerance to foreign innocuous antigens, such as those derived from the commensal microbiota. pTregs are shown to be generated *in vivo* and, indeed, mouse models could demonstrate non-redundant functions for pTregs supplementing tTregs [32,33], even though tTregs and pTregs share a common niche [34]. Currently there is no protein marker which can unambiguously distinguish tTregs from pTregs: the proposed tTreg marker Helios emerged to be not exclusively expressed in tTregs, and Neuropilin-1 (Nrp1) distinguishes murine but not human tTregs from pTregs under non-inflammatory conditions [31,35–37]. Interestingly, the TGF-β response element CNS1 in the Foxp3 locus appears crucial for peripheral pTreg differentiation while dispensable for tTregs [38], and its deletion in the C57BL/6 mouse strain exemplified the importance of pTregs *in vivo*, leading to spontaneous development of Th2-type pathologies at mucosal sites [39] as well as defects in maternal-fetal tolerance in the placenta [40].

Recently many factors contributing to Treg induction have been elucidated. TCR activation of naïve T cells in the presence of TGF-β and IL-2 favors differentiation of pTregs, which was shown in mice to occur *in vivo* and can be mimicked *in vitro* to generate so-called induced Tregs (iTregs). pTregs seem to play a role mainly in the intestine, where chronic low-dose antigen stimulation under tolerogenic conditions favors their induction. Gut-associated dendritic cells (DCs) do not only secrete TGF-β but also the vitamin A metabolite all-trans retinoic acid (ATRA), which enhances TGF-β-induced Treg induction [41–43]; also affecting human Tregs [44–46]. Further, it was recently shown that short-chain fatty acids (SCFA) derived from microbiota in the gut can induce murine Tregs *in vitro* and *in vivo* [47,48]. Along these lines, identifying factors inducing murine Tregs may aid in developing suitable protocols to generate human iTregs *in vitro* as well, which is highly relevant therapeutically. As Treg dysfunction is involved in many diseases, adoptive transfer of Tregs is suggested to be a promising strategy to prevent or cure autoimmune and inflammatory diseases. Importantly, adoptive transfer of *ex vivo* isolated Tregs to prevent or treat graft-*versus*-host disease (GvHD) showed promising outcomes in first-in-man clinical trials [49–52], and several other clinical studies are ongoing. Although controversial, several studies in mice indicate that the therapeutic transfer of iTregs may be superior to transfer of *ex vivo* isolated Tregs, rendering iTregs an attractive target for therapeutic approaches in humans as well [53]. This is particularly relevant as *ex vivo* derived Tregs are very limited in number and their expansion is not trivial [54]. Further, iTregs could be generated in an antigen-specific manner, thus enhancing efficacy and specificity. Numerous mouse studies show the effectiveness of *in vitro* generated iTregs in several disease settings [53], such as colitis, type 1 diabetes, autoimmune gastritis, arthritis and a model for multiple sclerosis. Further, *scurfy* mice, which exhibit severe systemic autoimmune disease due to Foxp3 mutation, can be rescued by transfer of wild type *in vitro* generated iTregs [55]. Of note, in xenogeneic GvHD models, transfer of human iTregs generated in the presence of TGF-β plus ATRA or plus Rapa successfully prolonged survival of mice [46,56,57]. However, therapeutic use of iTregs in humans requires further research on iTreg generation from human naïve T cells as it is currently highly disputed which protocols are suitable to generate human Tregs *in vitro*. Particularly, stability of iTregs is a concern, as it was suggested that Foxp3 expression in iTregs is unstable due to lack of TSDR demethylation [27]. For TGF-β-induced murine as well as human iTregs, most studies indeed find at most an intermediate demethylation pattern of the TSDR not comparable to tTregs and also correlating with intermediate suppressive activity [5,26,27,38,58,59]. Interestingly, *in vivo* generated pTregs acquire TSDR demethylation in most mouse models [24,27,59,60], but not in all [32]. Nevertheless, several studies find stable and suppressive human and murine iTregs without TSDR demethylation [56,61–63]. Adding to the controversies, even with similar protocols for iTreg generation, reports between laboratories differ with respect to phenotype and suppressive function of TGF-β-induced iTregs [64], which may be due to subtle experimental differences such as, for example, serum factors as well as type, strength and timing of stimulation [10,11,63]. Furthermore, the effect on human Foxp3+ Treg induction has not been investigated for several of the compounds recently described to favor murine iTreg generation.

To advance the knowledge of human Treg induction, here we comprehensively compared different proposed procedures for human iTreg induction side-by-side within the same experimental system, as well as developed new protocols for human iTreg induction. We characterized the derived human iTregs in detail with respect to their phenotype regarding Treg signature molecules, TSDR demethylation, cytokine production as well as suppressive function *in vitro* and *in vivo* in a humanized GvHD mouse model. Our results enhance the understanding of human iTreg generation and we identify the combination of TGF-β, ATRA and Rapa as a novel protocol to robustly induce human iTregs with superior suppressive activity *in vitro* compared to other protocols, yet stability and *in vivo* suppressive activity remain to be achieved in the future.

## Results

### Different protocols comprising TGF-β to induce human iTregs result in high Foxp3 expression

**Fig 1:**
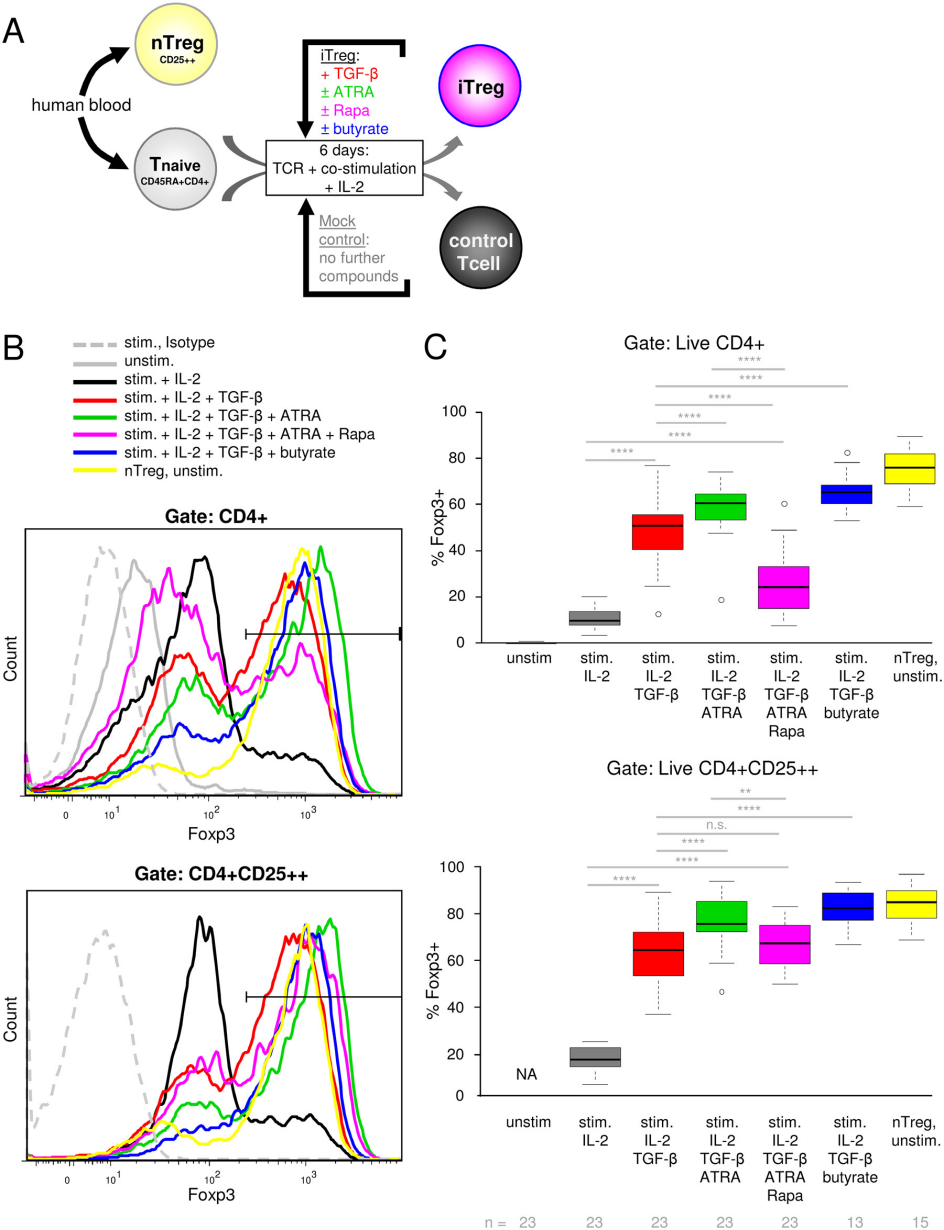
Foxp3 expression upon use of different protocols for human iTreg differentiation. (A) Experimental setup: Human naïve CD4 T cells were cultured for 6 days in serum-free medium under the indicated conditions. T cells were stimulated with anti-CD3 and anti-CD28 antibodies plus 100 U/ml IL-2 (“Stim.”). Where indicated, TGF-β1, rapamycin (Rapa), all-trans retinoic acid (ATRA) or butyrate were added. nTreg (*ex vivo* isolated peripheral blood CD25 high cells) were left unstimulated and used as positive control. (B) The histogram shows representative Foxp3 stainings for one donor, gated on CD4+ cells in the upper panel or on CD25++CD4+ cells in the lower panel. The striped line represents an isotype control staining (exemplarily shown for the condition “stim. + IL-2 + TGF- β + ATRA + Rapa“). Unstim. = unstimulated naïve T cells. (C) The boxplot shows percentages of Foxp3 positive cells measured by FACS as in (B) for n = 13 to 23 different donors in 8 to 13 independent experiments (n numbers are indicated below plot). The upper panel shows percentage of Foxp3+ cells from CD4+ cells, the lower panel of Foxp3+ cells from CD4+CD25++ cells. NA = not applicable. Significance was calculated by paired t test. n.s.: not significant, *: p<0.05; **: p<0.01; ***: p<0.001; ****: p<0.0001.

To analyze and compare the efficacy of different published and novel protocols to induce human iTregs, we isolated naïve CD4+ T cells from human peripheral blood and cultured them *in vitro* in the presence of TCR and costimulation plus IL-2 in serum-free medium for 6 days (Fig 1A and S1 Fig). Stimulated cells were used as a negative control (“mock”). To induce iTregs, TGF-β1 was added to the cultures, either alone or together with ATRA, ATRA plus Rapa, or butyrate. *Ex vivo* isolated CD25++ Tregs, here called nTregs, were used as positive control and presumably comprise a majority of tTregs, but also pTregs [31]. By comprehensively comparing these different protocols for human iTreg induction, we found that all of the tested protocols induced a high fraction of Foxp3 expressing cells, which was significantly higher than the weak activation-induced Foxp3 expression observed in human mock control T cells (Fig 1; S2 Fig; S1 Table). Foxp3 expression levels per cell in Foxp3+ iTregs were similarly high as in nTregs (Fig 1B). Foxp3 expression was enriched in the CD25++ population for all the TGF-β-induced iTregs, which was less obvious for activation-induced low Foxp3 expression in stimulated mock control cells (Fig 1B and 1C). We found that ATRA addition together with TGF-β enhanced Foxp3 induction compared to TGF-β alone. Conversely, when Rapa was added in addition to TGF-β and ATRA (termed “Rapa triple combination”), Foxp3 expression in total CD4+ T cells rather dropped compared to TGF-β or TGF-β + ATRA treatment alone. This seemingly reduced Foxp3 expression in the cultures containing Rapa was less pronounced when gating on CD25++ cells (Fig 1B and 1C). SCFAs were recently described to enhance Treg induction in murine cells [47,48], but the effect on human T cells was not studied yet. Here, we found that addition of butyrate together with TGF-β enhanced Foxp3 induction in human naive T cells compared to TGF-β alone. We found that propionate, another SCFA which was suggested to marginally enhance Treg induction in mouse cells [47,48], did not significantly enhance Foxp3 expression compared to TGF-β alone (data not shown). A similar trend of Foxp3 expression across the different iTreg conditions as shown in Fig 1 compiled for all donors was also seen in individual donors (S3A Fig), as well as on the level of *FOXP3* mRNA (S3B Fig). Kinetics as well as titration of TGF-β and anti-CD28 showed a similar trend of Foxp3 expression across Treg-inducing conditions regardless of the tested TGF-β or anti-CD28 concentrations, with our standard conditions (5 ng/ml TGF-β and 1 μg/ml anti-CD28) yielding the highest Foxp3 expression (S3C Fig). Though elevated concentrations of TGF-β are described to favor Treg over Th17 induction [65–67], increasing the TGF-β concentration to 10 ng/ml did not further enhance Foxp3 induction in our system, but 5 ng/ml were sufficient (S3C Fig). Since strong costimulation was suggested to inhibit Treg induction [10,15–17], and ATRA was proposed to increase Treg differentiation mostly in conditions of high costimulation [16], we tested lowering our anti-CD28 concentration. By doing so, we did not observe enhanced Foxp3 induction compared to our standard conditions, and the effect of ATRA enhancing Foxp3 induction was seen at both CD28 concentrations tested (S3C Fig). Together, these results show a distinct and reproducible pattern of Foxp3 induction in human T cells by different published (TGF-β; TGF-β + ATRA) and new (TGF-β + ATRA + Rapa; TGF-β + butyrate) protocols to induce human iTregs through direct comparison of these protocols within the same donors and experimental system.

### Human iTregs express other Treg signature molecules in addition to Foxp3

**Fig 2:**
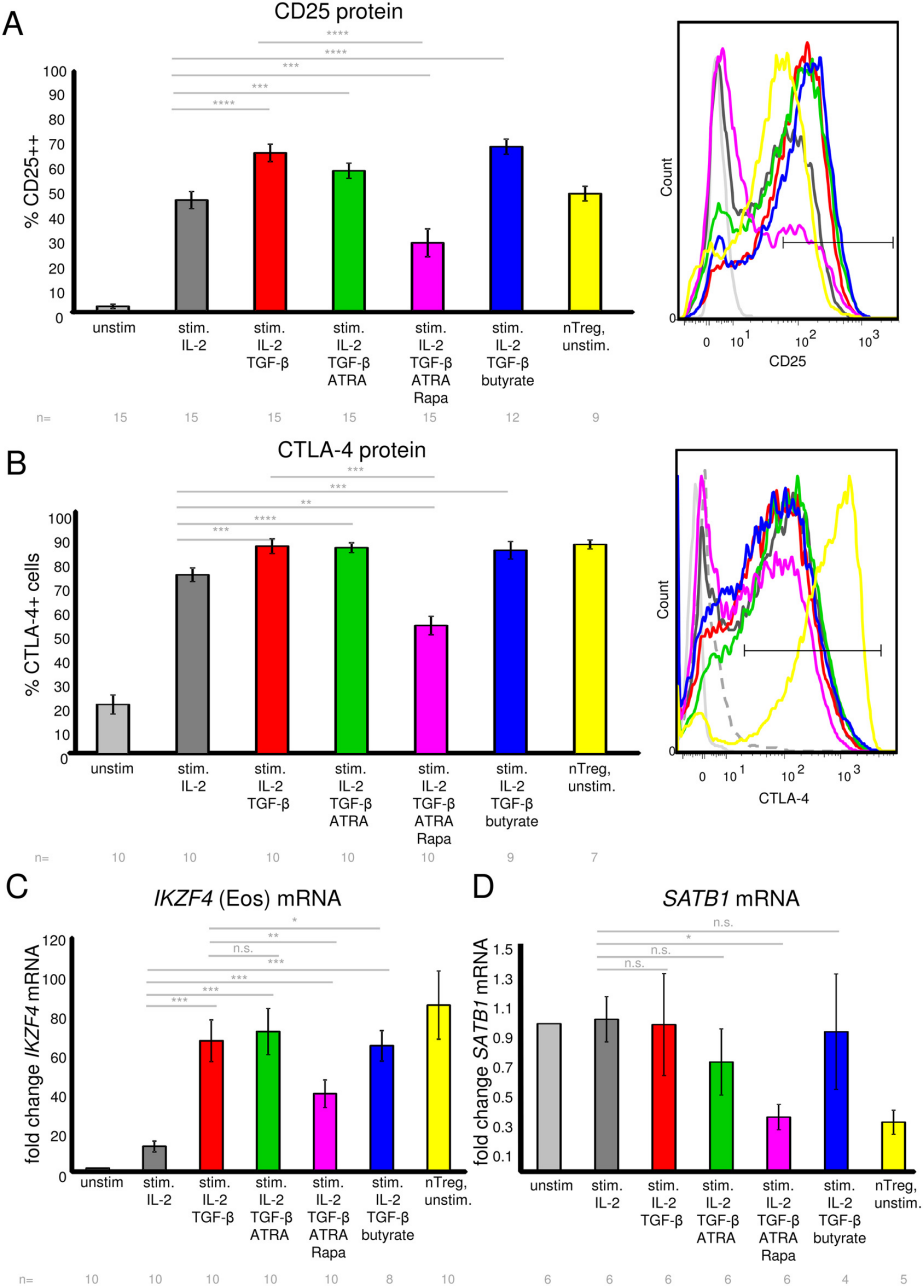
Expression of Treg signature molecules in human iTregs. (A) CD25 surface expression was measured by surface staining and flow cytometry on day 6 of culture under the indicated conditions, gated on live CD4+ cells. Shown are mean +/- SEM values for n = 9 to 15 donors (n number indicated below plot). Significance was calculated with paired t test. The right panel shows representative CD25 histogram overlays; color code as in the left panel. (B) CTLA-4 expression (surface and intracellular) was quantified by staining of permeabilized cells and flow cytometry on day 6 of culture under the indicated conditions, gated on live CD4+ cells. Shown are mean +/- SEM values for n = 7 to 10 donors. Significance was calculated with paired t test. The right panel shows representative CTLA-4 histogram overlays; color code as in the left panel. Dashed line represents CTLA-4 isotype control (isotype example shown for stim. + IL-2 + TGF-β + ATRA sample). (C) *IKZF4* (EOS) mRNA expression in naive T cells cultured 6 days under the indicated iTreg or control conditions. nTregs and unstimulated naive T cells were sampled on day 0. mRNA was quantified by Taqman assay, normalized to *RPL13A* expression. *IKZF4* mRNA expression in unstimulated naive T cells from the corresponding donor was set to 1, and fold change of *IKZF4* mRNA was calculated. Shown are mean +/- SEM values for n = 9to 10 donors. Significance was calculated with paired t test. (D) *SATB1* mRNA expression in naive T cells cultured 6 days under the indicated iTreg or control conditions. nTregs and unstimulated naive T cells were sampled on day 0. mRNA was quantified by Taqman assay as described in (C). Shown are mean +/- SEM values for n = 4 to 6 donors. Significance was calculated with paired t test. n.s.: not significant. *: p<0.05; **: p<0.01; ***: p<0.001; ****: p<0.0001.

Since Foxp3 expression alone is not sufficient to designate human Tregs, we sought to analyze expression of other Treg-like signature molecules and compare them between the different Treg-inducing protocols. The IL-2 receptor alpha chain CD25 and the coinhibitory molecule CTLA-4 are constitutively expressed by Tregs, but also by activated T cells. We found that iTregs generated by our protocols expressed high levels of CD25 and CTLA-4 in a large fraction of the population (Fig 2A and 2B; S4A Fig). Yet the expression was only marginally higher than in activated control T cells, except for the Rapa triple combination in which the fraction of CD25 and CTLA-4 expressing cells was rather slightly decreased. The latter effect was consistent with the decreased growth rate in the Rapa triple combination iTregs (unpublished observation) as well as the reduced fraction of Foxp3+ cells within the CD4, but not within the CD25 gate (Fig 1). CTLA-4 expression in iTregs did not reach the expression levels observed in nTregs (Fig 2B and S4A Fig). Since it was recently described for murine T cells that expression of Foxp3 together with either one of the so-called “quintet” transcription factors was sufficient to establish the full Treg-specific gene expression signature [6], we sought to analyze the expression of these quintet factors in our iTreg populations. The quintet factors IRF4, GATA-1 and EOS were described to be upregulated in Tregs, while SATB1 and LEF1 should be downregulated in Tregs; yet overexpression of each one of these factors together with Foxp3 in mouse T cells elicited the Treg signature [6]. We found that *IKZF4* (encoding for EOS) was highly expressed in all our iTregs at significantly higher levels than in naïve or activated control T cells, reaching levels in the range of nTregs (Fig 2C). Notably, EOS is known to be a crucial TF to ensure Treg phenotype, function and stability [68,69]. We found that *SATB1*, which is described to be downregulated in Tregs [6] with functional relevance [70], was significantly downregulated in iTregs induced by the Rapa triple combination, which showed expression levels comparably low to nTregs (Fig 2D). For other iTreg protocols, *SATB1* downregulation was less pronounced. Regarding the other “quintet” factors, we did not find *IRF4* specifically expressed in iTregs nor nTregs, and also, *LEF1* was not downregulated in iTregs (S4B and S4C Fig). Expression of *GATA-1* was generally very low and close to detection limit (Ct values range 32 to 37 or undetermined, n = 4 donors; S1 Table) and was therefore not analyzed further.

### Human iTregs display low IFN-γ, IL-17 and IL-10 cytokine expression

**Fig 3:**
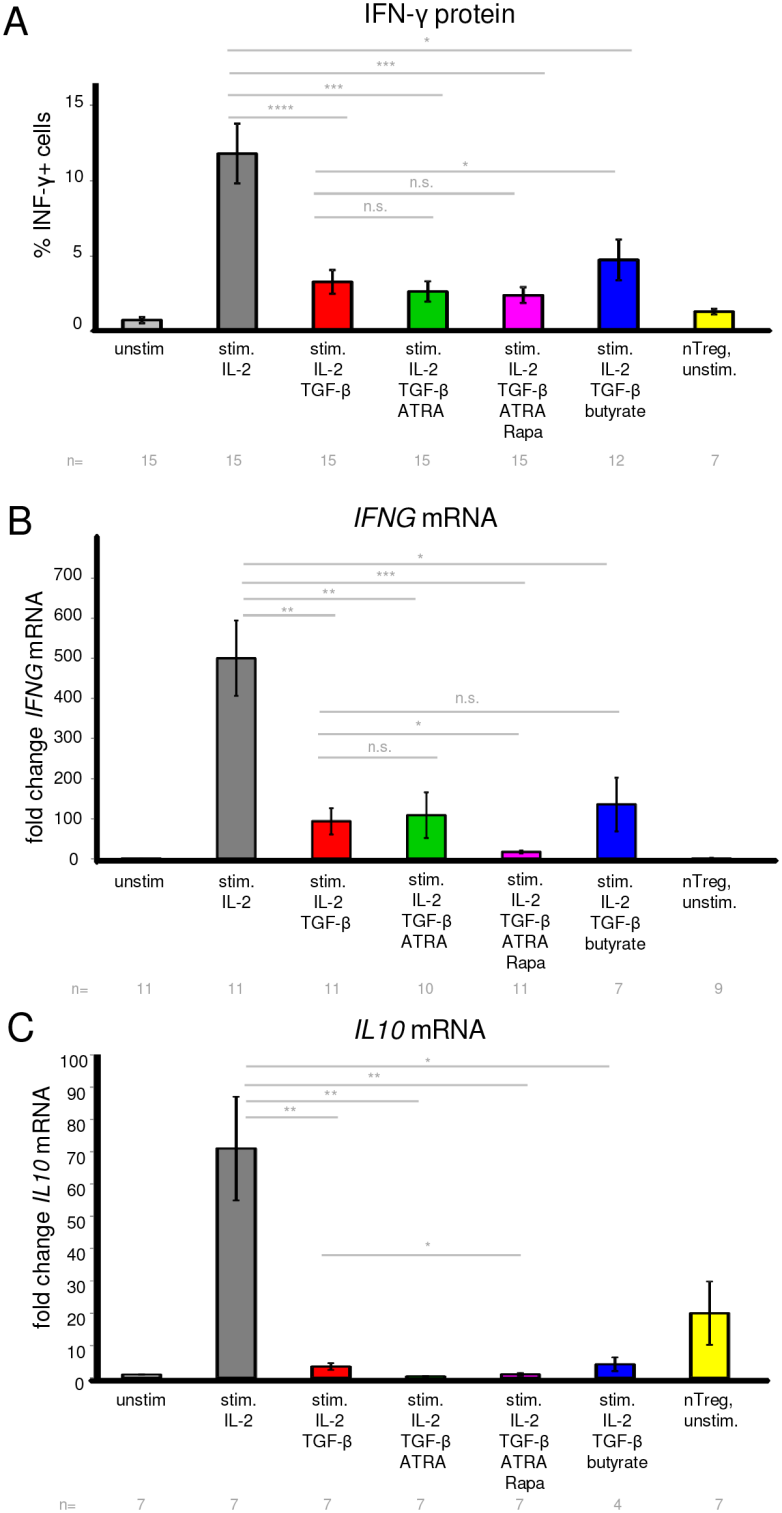
Cytokine production by human iTregs. (A) IFN-γ expression was measured by intracellular staining after 4 hours of PMA/ionomycin pulse on day 6 of culture under the indicated conditions, gated on live CD4+ cells. Shown are mean +/- SEM values for n = 7 to 15 donors (n numbers indicated below the bar chart). Significance was calculated with paired t test. (B) *IFNG* mRNA expression in naive T cells cultured 6 days under the indicated iTreg or control conditions. nTregs and unstimulated naive T cells were sampled on day 0. mRNA was quantified by Taqman assay, normalized to *RPL13A* expression. *IFNG* mRNA expression in unstimulated naive T cells from the corresponding donor was set to 1, and fold change of *IFNG* mRNA was calculated. Shown are mean +/- SEM values for n = 7 to 11 donors (n numbers indicated below the bar chart). Significance was calculated with paired t test. (C) *IL10* mRNA expression in naive T cells cultured 6 days under the indicated iTreg or control conditions. nTregs and unstimulated naive T cells were sampled on day 0. mRNA was quantified by Taqman assay as described in (B). Shown are mean +/- SEM values for n = 4 to 7 donors (n numbers indicated below the bar chart). Significance was calculated with paired t test. n.s.: not significant. *: p<0.05; **: p<0.01; ***: p<0.001; ****: p<0.0001.

One concern about iTregs as opposed to nTregs is that they might express high levels of pathogenic inflammatory cytokines, such as IFN-γ or IL-17, which could be detrimental for therapeutic use in adoptive transfer. We found that iTregs generated by the different protocols described above expressed only low levels of IFN-γ when measured on protein level intracellularly after PMA/ionomycin restimulation (Fig 3A) or without restimulation on mRNA level (Fig 3B). IFN-γ expression was strikingly lower in iTregs compared to stimulated mock control cells, with lowest expression in iTregs generated with the Rapa triple combination. Addition of butyrate seemed to slightly hamper IFN-γ repression (Fig 3A and 3B). iTregs and Th17 cells are closely related and share common pathways during differentiation, yet have opposing functions with IL-17 having mainly pro-inflammatory roles [71]. Thus we analyzed whether iTregs induced by our protocols also led to enhanced IL-17 production, but we could not detect substantial amounts of IL-17A in the iTreg populations tested (qRT-PCR Ct values range 32–37 or undetermined; intracellular staining range 0.01 to 0.7% IL-17A+ cells, n = 4–7 donors; S1 Table). The immunosuppressive cytokine IL-10 is one potential mechanism by which iTregs or other regulatory cell populations can suppress immunity [72], which is why we investigated IL-10 expression in the different iTreg inducing conditions. IL-10 expression was lower in iTregs compared to stimulated control T cells or nTregs (Fig 3C), arguing against an important role for IL-10 in the iTreg populations under study.

### Foxp3 expression is fairly stable upon short-term resting in IL-2 containing medium

**Fig 4:**
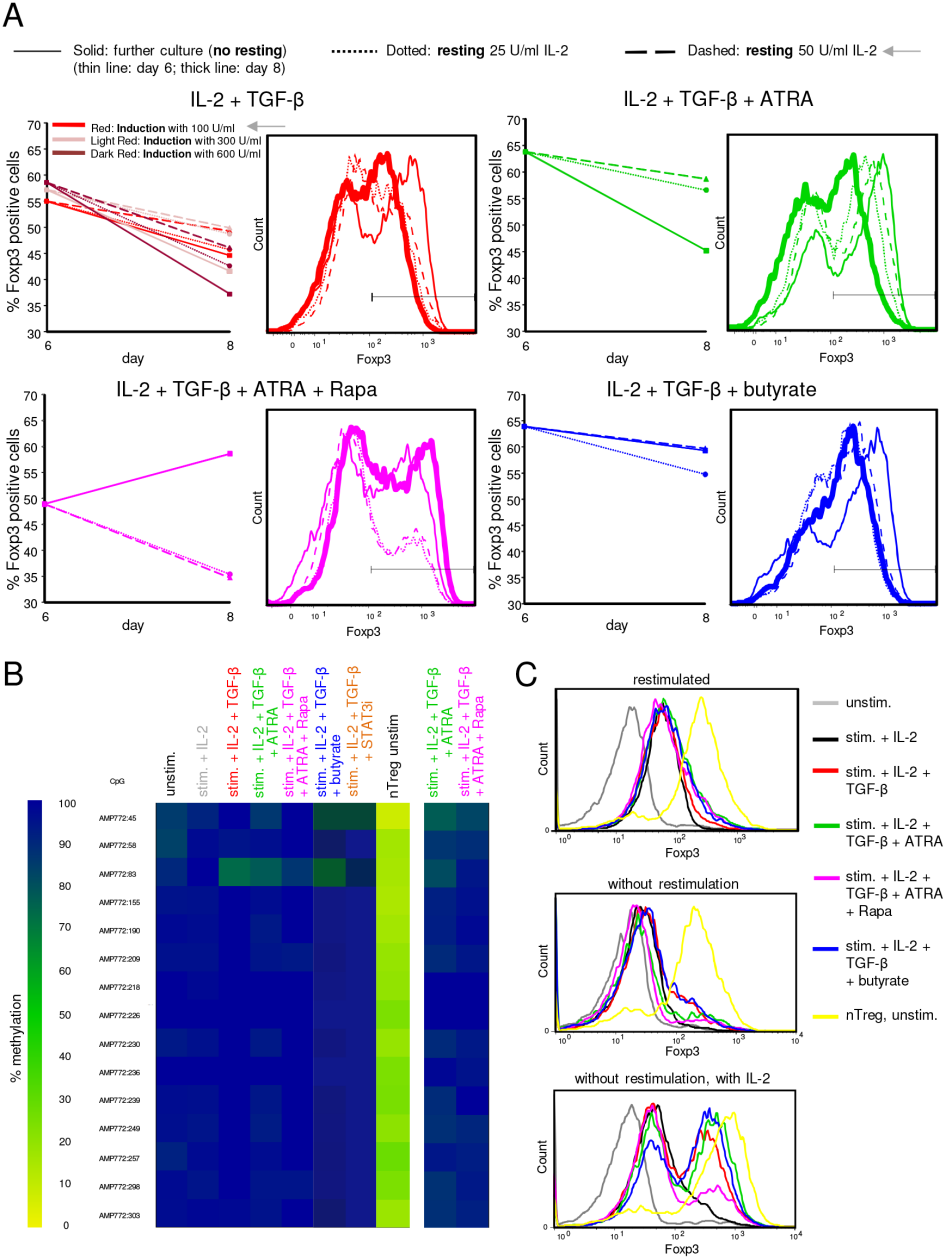
Analysis of Foxp3 stability and TSDR demethylation in human iTregs. (A) iTregs were induced for 6 days under the indicated conditions, with 100 U/ml IL-2 if not otherwise stated. Foxp3 protein expression on day 6 is shown as solid, thin line. After 6 days, iTregs were either further cultured 2 days (thick line) or washed and rested in medium containing only IL-2, either 25 U/ml (dotted lines) or 50 U/ml (dashed lines). Left panels show the percentage of Foxp3+ cells, right panels show FACS histogram overlays for corresponding Foxp3 stainings. The “standard”conditions (100 U/ml IL-2 for induction, 50 U/ml IL-2 for resting) are marked by grey arrows. Gate: Live CD4+ cells. Shown is a representative donor (out of 4 to 6 for standard conditions; out of 2 for IL-2 titrations during induction). (B) TSDR DNA demethylation was analyzed at day 6 of Treg induction under the indicated conditions (left panel), or on day 8 after resting (two lanes on the right). Tnaive and nTreg DNA was isolated on day 0 as control. T cells were isolated from a male donor, and a representative donor out of 2 to 4 is shown. The color scale indicates 0 to 100% methylation at the given CpG nucleotides in the Foxp3 locus. (C) iTregs or control cells were cultured for 6 days under the indicated conditions, then rested 2 days, and further cultured for 5 days with restimulation, or without restimulation and without or with IL-2 (upper, middle and lower panel, respectively). nTregs were cryopreserved after isolation, thawed on day 6 and rested 2 days, and then treated in parallel with iTregs as a control. Intracellular Foxp3 expression was measured by flow cytometry, gated on live CD4+ cells. Shown is a representative donor out of 4.

Stability of Foxp3 in iTregs is controversial and has not been investigated under the exact conditions used here, thus, we addressed the question whether Foxp3 expression in iTregs was stable upon resting the cells without Treg-inducing additives. Furthermore, Tregs should be rested without stimulation and Treg-inducing compounds before analyzing their suppressive function (see below), so we sought to establish and characterize resting conditions for iTregs. After 6 days of Treg induction, iTregs were either further cultured for 2 days, or washed and rested for 2 days in medium containing only low concentrations of IL-2 (S5A Fig). We found that for most protocols, Foxp3 expression was slightly decreasing from day 6 to day 8 when iTregs were further cultured under iTreg-inducing conditions (Fig 4A). When iTregs were instead washed on day 6 and rested 2 days, 50 U/ml IL-2 led to marginally better maintenance of Foxp3 expression compared to 25 U/ml IL-2 (Fig 4A). Even when initial Foxp3 expression was enhanced by adding higher IL-2 concentrations during the 6 days induction phase, this enhancement of Foxp3 expression was no more obvious after the resting phase (Fig 4A, upper left panel). Also on mRNA level, *FOXP3* expression was reduced after the resting period (S5B Fig). Together, the results demonstrate that Foxp3 expression was slightly lost after short-term resting of iTregs in low concentrations of IL-2, but the trend of Foxp3 expression across the different Treg-inducing conditions was maintained after resting on day 8. TGF-β addition during the resting phase did not rescue the minor loss of Foxp3 expression (S5C Fig), but rather slightly decreased viability (data not shown). Therefore, we chose resting with 50 U/ml IL-2 and without TGF-β as standard condition for further experiments.

An important marker for stable Foxp3 expression is demethylation of the TSDR in the Foxp3 locus, the existence and necessity of which is controversial for iTregs and has not been tested for most of those iTreg-differentiating protocols used here. Therefore, we asked whether iTregs in this study displayed TSDR demethylation. As a result, we could not detect TSDR demethylation in any of the iTreg populations on day 6 (Fig 4B), which was also the case for iTregs generated in the presence of TGF-β plus a STAT3 inhibitor recently proposed to enhance TSDR demethylation [73]. Establishment of TSDR demethylation might potentially take longer than 6 days in our system, yet those iTregs analyzed on day 8 after resting still did not display TSDR demethylation (Fig 4B). Together, we found that despite high Foxp3 expression, none of the iTregs generated by different protocols displayed Foxp3 TSDR demethylation, yet Foxp3 expression was relatively stable at least during short-term resting.

### Foxp3 is maintained without restimulation in the presence of IL-2, but not upon restimulation

So far, we only tested Foxp3 stability upon short-term resting, which resulted in relatively stable Foxp3 expression. However, particularly in light of absent TSDR demethylation, we tested whether iTregs could maintain Foxp3 expression when restimulated, or kept without restimulation for a longer period in culture (S6A Fig). We found that upon restimulation for further 5 days, iTregs generated by either protocol lost Foxp3 expression to nearly undetectable levels (Fig 4C, upper panel), even though cell viability remained high (S6B Fig). Also without restimulation, iTregs largely lost Foxp3 (Fig 4C, middle panel). Moreover, under such conditions without restimulation, a major fraction of iTregs died (S6B Fig). However, when iTregs were instead further cultured for 5 days without restimulation and supplemented with IL-2, cell death was prevented (S6B Fig) and cells largely retained their Foxp3 expression (Fig 4C, lower panel). Yet, even at this later time point under these latter conditions with stable Foxp3 expression, we could still not detect TSDR demethylation (S6C Fig).

### Foxp3 re-induction enhances Foxp3 stability, but does not induce TSDR demethylation

Next, we asked whether Foxp3 expression could be maintained more stably if during a longer resting phase for about another week the medium was supplemented again with Treg-inducing molecules such as TGF-β (S7A Fig). Indeed, we found that compared to resting in IL-2 containing medium alone, re-inducing iTregs with the initially added TGF-β + ATRA or TGF-β + ATRA + Rapa led to a better maintenance of Foxp3 expression (S7B Fig). Yet, this re-induction for a longer period was still not accompanied by TSDR demethylation (S7C Fig).

### iTregs induced with TGF-β plus ATRA plus Rapa display robust suppressive function *in vitro* but not *in vivo*

**Fig 5:**
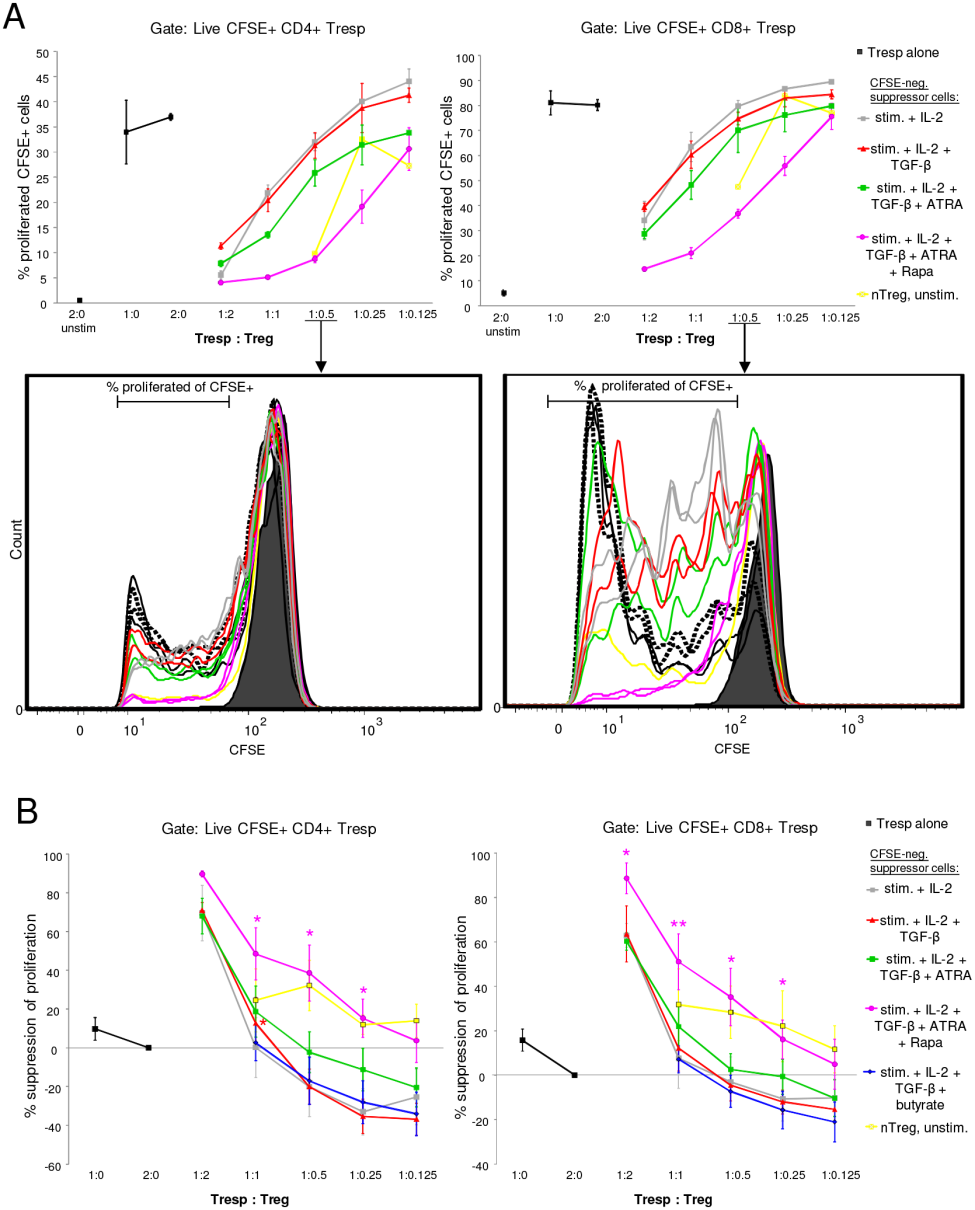
Comparative analysis of *in vitro* suppressive function of human iTregs generated with different iTreg-inducing protocols. iTregs were induced 6 days under the indicated conditions, washed, rested 2 days, washed again, and then used to analyze their suppressive capacity towards Tresp. Suppression assays with iTregs or control cells were performed with CFSE-labeled CD3+CD25- Tresp. Tresp and iTregs (or control stimulated mock suppressor cells, grey line) were cocultured at the indicated ratios, or Tresp cultured alone (black) in different densities. nTregs (yellow) were used as control. Cultures were stimulated for 4 to 5 days and then analyzed by FACS. (A) shows a representative donor. It was gated on CFSE+ CD4+ Tresp (left panel) or CFSE+CD8+ Tresp (right panel). The upper plots show mean +/- SD of wells plated in replicate. The lower panel shows overlays of CFSE histograms for the 1:0.5 ratio. Unstim. = unstimulated; others are stimulated for 5 days with pb anti-CD3 and soluble anti-CD28 antibodies. Dotted black lines = 2:0 Tresp, solid black lines = 1:0 Tresp, filled histogram = unstimulated Tresp. Lines in same color represent cells plated in replicate wells. The gate for determining the percentage of proliferated cells is indicated by the horizontal line. (B) Shows percent suppression calculated from CFSE proliferation as in (A), with 2:0 Tresp set to 100% proliferation (0% suppression). Shown is the compiled data (mean +/- SEM) for n = 2 to 6 donors (n = 2 for 1:2 ratio; n = 4 for “butyrate”iTreg, all other iTregs: n = 6 donors). Significance was calculated by paired t test, comparing suppression by iTreg populations to mock suppressor cells (stimulated with anti-CD3/-CD28 and IL-2 only; grey line) within each donor at 1:1; 1:0.5 or 1:0.25 cell ratios. Asterisks indicate significant differences and are depicted in the color of the respective iTreg condition. *: p<0.05; **: p<0.01.

Even though much progress has been made over the past decade to delineate Treg signature molecules, there is still no definite marker which unequivocally defines Tregs. Thus, it is crucial to determine their suppressive function. Regarding suppressive activity of iTregs, reports are contradictory, leaving it unclear whether TGF-β induced Foxp3 expression is sufficient to elicit suppressive activity. Further, even though some studies have compared different protocols to induce human Tregs, there is no comparative study analyzing suppressive function of the iTregs generated by all the different protocols as we apply here. Since it is difficult to compare the activity of iTregs generated by diverse protocols across different laboratories and types of suppression assays, we sought to compare all Treg-inducing protocols described here side-by-side in the same experimental system regarding their ability to induce suppressive iTregs, compared to stimulated mock suppressor cells from the same donor. In contrast to assays with nTregs which are anergic *in vitro*, for suppression assays with iTregs it has to be considered that iTregs are pre-activated, proliferative *in vitro* and may secrete a range of cytokines. Therefore, to analyze their suppressive function, we rested iTregs and then cocultured them in different ratios with CFSE-labeled responder T cells (Tresp; S8 Fig). Mock suppressor cells treated in the same way were used as control, as well as previously frozen nTregs from the same donor. Subsequently, suppression of proliferation and intracellular IFN-γ production in Tresp was analyzed by gating stringently on Tresp and, importantly, live cells (S9 Fig). In our experimental system, we found that only iTregs induced by the TGF-β + ATRA + Rapa triple combination specifically and consistently suppressed responder CD4 and CD8 T cell proliferation at a range of different Tresp:iTreg ratios and better than all other iTreg conditions tested (Fig 5 and S10A Fig). For the other iTreg populations, even though some suppression was observed at high iTreg:Tresp ratios, this did not seem to be specific to Foxp3+ iTregs since activated mock suppressor cells at higher ratios inhibited Tresp proliferation to a similar extent in some donors (Fig 5 and S10A Fig). Also in suppression assays containing antigen-presenting cells, Rapa triple combination-induced iTregs were the best suppressive iTregs (S10B Fig). Along those lines, TGF-β + ATRA + Rapa triple combination-induced iTregs were also superior in inhibiting IFN-γ production of Tresp compared to other iTreg populations or mock suppressor cells, though donor variability was relatively high here (S10C Fig).

Together, our *in vitro* results show that only TGF-β + ATRA + Rapa triple combination-induced iTregs suppressed responder T cells significantly, thus identifying this combination as a new robust protocol to induce iTregs with specific and superior suppressive activity *in vitro* compared to all other iTreg differentiation protocols tested.

**Fig 6:**
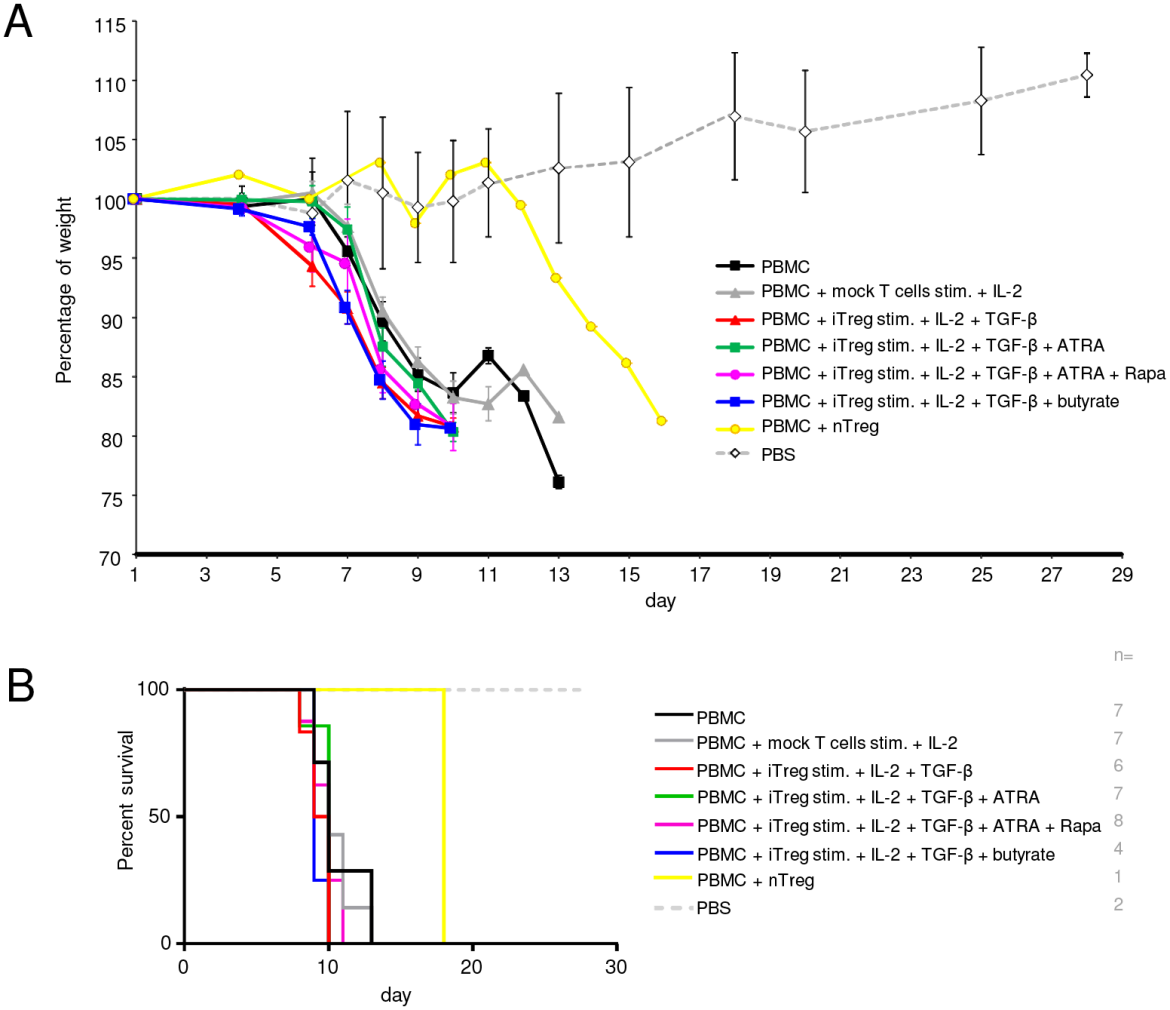
Analysis of suppressive function of human iTregs *in vivo* in xenogeneic GvHD. iTregs were induced 6 days under the indicated conditions, then washed and rested for 2 days. Allogeneic PBMCs were isolated and CD25-depleted the day before injection. NOG mice were irradiated and injected intravenously on the same day with 20x10^6^ PBMCs alone or together with 5x10^6^ iTregs or mock control T cells or expanded nTregs in PBS. As control, irradiated mice received PBS only. Mice were weighed every 1 to 3 days. (A) Shows percentage of mouse weight as mean +/- SEM for n = 4 to 8 mice per iTreg group combined from 2 independent experiments; mouse numbers (n) are indicated in the figure (B). (B) Shows the corresponding survival curves for the data from (A). There were no significant differences when comparing each iTreg group to either PBMC only or mock T cell groups.

Since *in vitro* suppression assays do not necessarily reflect the situation *in vivo*, and we found that iTregs lost Foxp3 upon restimulation (Fig 4C), we asked whether iTregs generated by the TGF-β + ATRA + Rapa triple combination were also suppressive *in vivo*. To this end, we used a xenogeneic GvHD model, in which human PBMCs induce GvHD upon injection into immunodeficient NOG mice [74]. In addition to the demonstration of protective effects of nTregs on GvHD development in this model, it has also been used to show *in vivo* suppressive function of certain iTreg populations [46,56,57]. We found that GvHD was rapidly induced by intravenous injection of PBMCs into irradiated NOG mice as monitored by weight loss and survival. However, iTregs generated by any of the tested protocols were not able to prevent or delay GvHD (Fig 6). Thus, while iTregs generated by combining TGF-β + ATRA + Rapa had superior suppressive activity *in vitro*, this was not accompanied by *in vivo* suppressive function in a xenogeneic GvHD model.

## Discussion

The impact of Tregs on human health is well-documented, provoking considerable interest from the pharmaceutical community to target Treg development, transfer and function for a range of diseases, predominantly autoimmune and inflammatory diseases as well as cancer. Not only may the function or differentiation of endogenous Tregs be manipulated, but also the adoptive transfer of Tregs is increasingly becoming a realistic option for clinical routine. First in-man trials achieved promising outcomes with adoptive Treg transfer in preventing or treating GvHD after allogeneic stem cell transplantation [49–52]. It is possible that depending on the disease, transfer of nTregs or iTregs would be more suitable contingent on their stability *in vivo* in the respective cytokine milieu, their location, and on which suppression mechanism they utilize. Further, since the number of nTregs is minute and their expansion intricate, iTreg transfer appears as an attractive concept for therapeutic approaches. In this regard, *in vitro* iTreg generation needs to be improved in terms of efficacy, specificity, and safety. Much of the basis for our current knowledge on Tregs has been derived from mouse models, while less is known about primary human T cells. Yet differences between mice and humans manifest also with respect to Foxp3 expression. As several mouse studies suggest superior therapeutic effects of iTreg compared to nTreg transfer [53], it is important to further clarify the conditions for human iTreg differentiation in light of immunotherapy approaches.

Here, we comprehensively compared several protocols described to induce human iTregs, as well as tested several new protocols such as those described so far only for murine iTregs. The tested protocols included not only TGF-β, but also ATRA, Rapa, and butyrate. We could confirm results from previous studies, which showed that addition of ATRA enhanced human Foxp3 induction compared to TGF-β alone [44–46]. It was also suggested that Rapa could enhance TGF-β-mediated Treg induction, initially described in mouse cells [20] and subsequently confirmed in human cells [56,57]. Due to these described effects of ATRA or Rapa separately, and since ATRA and Rapa may act synergistically in maintaining the Treg phenotype as shown for human Treg expansion [75,76], we asked whether their combination, together with TGF-β, would also improve human iTreg induction. Indeed, we found that this triple combination of TGF-β + ATRA + Rapa, which is novel to generate human iTregs, generated iTregs which displayed the most suppressive activity *in vitro* (see below) even though not further enhancing Foxp3 induction and not suppressing GvHD *in vivo*. Based on mouse studies, it was suggested that ATRA and Rapa each contribute specific migratory capacities to iTregs *in vivo* [16,41,44,77–79], which may be advantageous for their suppressive capacity *in vivo* even though the latter was not tested for the triple combination [79]. In contrast to our results with human cells, however, this recent mouse study [79] did not observe a reduced fraction of Foxp3+ cells when combining ATRA and Rapa in TGF-β mediated Treg induction compared to cultures without Rapa; the reason for this discrepancy remains unclear and may be related to differences in species or experimental setup. One important aspect regarding the latter is the strength, kinetics and duration of TCR signaling: Several studies have shown that suboptimal, weak T cell activation promotes Foxp3 induction [10,12,15,18,21]. Indeed, later studies have confirmed that for initial Foxp3 induction, weak TCR stimulation (that is suboptimal for proliferation) favored Foxp3 induction [11]. However, in contrast to Foxp3+ cells induced by weak TCR agonists, only Tregs induced by a low density of a strong TCR ligand were stable and persistent over time in mice [10,11]. It is possible that in our study, Rapa blunted the effect of strong TCR stimulation (by plate-bound anti-CD3 antibodies) through inhibition of Akt and, therefore, contributes to the enhanced *in vitro* suppressive activity of TGF-β + ATRA + Rapa triple combination iTregs. Nevertheless, Gottschalk *et al*. demonstrated that despite triggering similar levels of Akt phosphorylation, high doses of weak agonist TCR ligands could not generate persistent Foxp3 induction as opposed to stable Foxp3 induction by low density of strong agonist ligands [11].

Recently high interest has been raised in the effect of intestinal commensal microbiota-derived metabolites such as SCFAs. It was recently shown that colonic Tregs isolated from mice treated with the SCFA propionate showed enhanced Foxp3 expression [80], yet it remained possible that indirect effects on Treg induction *via* DCs or macrophages [81], as well as additional effects on tTregs, contribute to this effect. Other studies [47,48] demonstrated minor effects of propionate and strong effects of butyrate on Treg induction *in vivo*, as well as direct effects on iTreg differentiation *in vitro*, enhancing TGF-β-induced Foxp3 expression in murine iTreg populations. Effects of SCFAs on human iTregs have not been studied to our knowledge and indeed, we could confirm that 0.1 mM butyrate enhanced TGF-β-mediated Foxp3 expression in human iTreg populations (Fig 1), while propionate at the same concentration did not have an effect (data not shown). Of note, at higher concentrations (0.25 mM), we observed that butyrate-treated iTregs displayed enhanced IFN-γ production compared to those treated with TGF-β only (data not shown), in line with the general function of butyrate as histone deacetylase inhibitor [82] as well as a recent report suggesting that SCFAs can promote T cell differentiation into both effector and Treg lineages depending on the surrounding milieu [83], which should be considered when using SCFAs in Treg induction.

Apart from Foxp3, our study also systematically compares the iTreg phenotype derived from all different iTreg-inducing protocols regarding other Treg signature molecules as well as cytokine expression of the generated iTregs. Thus, our study expands the previous knowledge on the phenotype of human iTregs and shows that all iTreg populations express significantly more *IKZF4* (encoding for EOS) and less IFN-γ compared to stimulated mock control cells, while CD25 and CTLA-4 expression showed only modest differences compared to the mock control. Interestingly, we could not find iTreg-specific expression of the proposed Treg “quintet” factors *IRF4*, *LEF1* and *GATA-1* [6], and it remains subject to future investigations whether this is based on human *versus* mouse differences. *SATB1*, another quintet factor, was however downregulated only in Rapa triple combination iTregs to a similar extent as in nTregs, potentially highlighting an important role for this factor in human iTregs.

However, despite all phenotypic characterization, it is indispensable to test Treg suppressive function in order to ascertain that Treg-like cells are generated, because Foxp3 expression alone seems insufficient to ensure Treg function. Also, species differences exist regarding Foxp3 induction. Particularly human iTreg suppressive function is accompanied by many controversies, to which the transient low expression of Foxp3 in human activated Tcons in part contributes. To date, contradictory reports leave it unclear whether (temporary) Foxp3 expression is sufficient to confer suppressive abilities to human activated Tcons. Some studies demonstrated Treg phenotype or suppressive capacities of Foxp3+ activated human T cells [3,84] while others did not [4,85,86]; yet others did not observe activation-induced transient Foxp3 induction in human Tcons [87]. These discrepancies might be due to differences in culture conditions, such as strength of TCR stimulation and presence of serum factors, IL-2 or TGF-β. To have a defined experimental system excluding serum factors, we used serum-free medium throughout this study. On a similar note, Shevach’s group found that TCR-induced Foxp3 expression in human naïve Tcons is indeed dependent on TGF-β, but does not result in a suppressive phenotype [88]. Conflicting results may also be ascribed to differences in the experimental readout used, as well as in kinetics and level of Foxp3 expression and, consequently, Foxp3 target genes [89–91] such as CTLA-4 and CD25. In the present study, we observed a minor activation-induced Foxp3 expression upon culture in serum-free medium without TGF-β addition, yet the fraction of cells expressing Foxp3 at levels comparable to nTregs remained very small (~10%). Still, when used at high ratios, we observed some suppressive activities of activated mock suppressor T cells towards responder T cells *in vitro*, which might be dependent or independent of Foxp3, and could be related to cell density effects, IL-2 consumption by CD25 or inhibitory effects of CTLA-4. Therefore, we consider it important, particularly when analyzing iTregs which are strongly pre-activated and proliferative, to include such a mock suppressor cell control in suppression assays. Along similar lines, others have also reported nonspecific suppression by stimulated Foxp3-negative T cells [10,92–94]. When comparing to this control, we found that, even though a misleadingly cell ratio-dependent inhibitory activity seemed to be present, only the Rapa triple combination-iTregs but not other iTreg populations suppressed significantly more than the mock suppressor cells *in vitro*. This is in contrast to a recent study on mouse cells [79], in which all iTregs, whether generated with TGF-β, TGF-β + ATRA or TGF-β + ATRA + Rapa, suppressed equally *in vitro*. It remains to be analyzed whether this divergence is due to differences in species or experimental setup, such as stimulation conditions or lack of a mock suppressor cell control. We think that lack of this mock suppressor cell control, as well as differences in strength of TCR stimulation, might add to controversies observed in the literature regarding suppressive function of human iTregs. Therefore, we consider it important to compare different Treg-inducing protocols side by side within the same experimental system and readout, such as in the present study in which we extensively compare many different protocols. Along these lines, although several studies show suppressive function of TGF-β-induced Foxp3+ human iTregs, some studies did not observe suppressive function of these cells. For example, Tran *et al*. [88] found that TGF-β induces Foxp3 but not suppressive function in human T cells, however, they neither tested additional compounds such as ATRA nor did they test *in vivo* suppressive function. Later studies [44–46,56–57] instead have shown that addition of ATRA or Rapa on top of TGF-β can dramatically change iTreg properties, leading to acquisition of suppressive function when compared to iTregs induced with TGF-β only—however, the TGF-β + ATRA + Rapa triple combination as well as TGF-β + butyrate have not been studied in human T cells before. Of note, not only the suppressive function of TGF-β-induced human iTregs is controversial, but also the proposed establishment of suppressive activity by accessory addition of ATRA [44–46] or Rapa [56,57] has been debated [64]. Nevertheless, although *in vitro* induction of human iTregs led to controversial results regarding suppressive function, recent studies show that by application of suitable protocols, these cells can effectively suppress *in vitro* and *in vivo* [53]. For example, Lu *et al*. [46] found that human iTregs generated with ATRA plus TGF-β suppressed better *in vitro*, and in contrast to iTregs generated with TGF-β only, they also suppressed *in vivo* in a xenogeneic GvHD model, even showing higher stability than nTregs in a proinflammatory cytokine milieu. Another study also suggests increased suppressive activity of human iTregs generated with TGF-β plus ATRA, yet TGF-β only also led to a, though less, suppressive population [44]. Two other studies [56,57] found that, compared to TGF-β only, adding Rapa during Treg induction led to iTregs with superior suppressive activity, even *in vivo* in xenogeneic GvHD models. However, in our study we did not observe suppressive activity of iTregs generated by either protocol when tested *in vivo* in xenogeneic GvHD. These controversial results may be related to differences in type and strength of TCR stimulation used in the different studies, which can influence stability of Foxp3 and, thus, suppressive potency in long-term assays [10,11]. While others [46,56,57] used bead-bound anti-CD3/CD28 antibodies or artificial APCs to induce Tregs, we have used plate-bound anti-CD3 and soluble CD28 antibody. Importantly, a very recent study [63] on murine iTregs found that only iTregs induced by bead-bound anti-CD3/CD28 antibodies—and not by plate-bound CD3/CD28 antibodies—were stable for >7 days *in vitro* and *in vivo* and, consequently, could suppress murine GvHD *in vivo*. Interestingly, this increased stability of murine iTregs generated by bead-bound stimulatory antibodies was not accompanied by decreased TSDR methylation [63]. This comparison of bead-bound *versus* plate-bound antibody-mediated stimulation for murine iTreg generation was suggested to resolve controversies regarding *in vivo* suppressive activity of murine iTregs used by Gu *et al*. [63] in contrast to others [95,96] who have employed similar murine GvHD models (but have used different modes of iTreg stimulation) and did not observe suppressive ability of different murine iTregs. It remains to be determined in future studies how quality and strength of TCR stimulation affect human iTreg stability and function. Affinity, duration and density of TCR stimulation were shown to influence stability of Foxp3 in murine iTregs also in other settings, in that only Foxp3+ cells induced by low doses of strong TCR agonists displayed stable Foxp3 expression whereas those induced by weak agonists were deleted [11]. It is therefore likely that modulating the TCR signal strength can also be used to optimize stability of human iTregs in the future.

It remains subject to future investigations to determine the reason for the discrepancy regarding suppressive activity of Rapa triple combination-iTregs in *in vitro* compared to *in vivo* assays. One cause might be loss of Foxp3 expression upon strong restimulation of iTregs *in vivo* in GvHD, similar to the loss observed upon *in vitro* restimulation (Fig 4). While xenogeneic GvHD models are the current tool of choice to enable experimental studies of *in vivo* suppressive activity of human (i)Tregs, it has to be considered that xenogeneic responses are usually stronger than allogeneic responses, more difficult to control, and occurring under shortage of human APCs—hence these models appear somewhat artificial [97–99]. Besides xenogeneic GvHD, other humanized models (such as humanized mouse models of skin or islet allograft rejection [100,101]) have been developed to study human Treg function *in vivo* and might be useful in the future to determine iTreg suppressive abilities.

However, an inherent limitation of all these models is the lack of human IL-2 production in immune deficient mice. Therefore, another reason underlying the discrepancy of results from *in vitro* and *in vivo* suppression assays in our study that remains to be investigated may be death of iTregs *in vivo*, such as observed *in vitro* without restimulation when IL-2 levels were limiting (Fig 4; S5 Fig). Others [46] have suggested that the lack of human IL-2 production in NOG mice leads to the non-permanent nature of protective effects of iTregs in xenogeneic GvHD models. Notably, even nTregs do not protect permanently in xenogeneic GvHD models which may be caused by lack of IL-2, which is well-known to be required for Treg maintenance and function [102–105]. Furthermore, it cannot be excluded that iTregs die *in vivo* under GvHD conditions due to other reasons, such as for example killing by strongly activated CD8+ T cells. In case of putative death of iTregs *in vivo*, the potential suppressive activity of the cells cannot be properly addressed, which needs to be considered when interpreting *in vivo* suppression assays.

Besides the stimulation methods applied, the addition of Treg-inducing compounds such as ATRA, Rapa, *etc*. is important for optimal iTreg generation. Considering our results in light of these aspects and along with studies of homing markers on mouse iTregs [79], we propose that our newly described protocol using a combination of TGF-β, ATRA and Rapa which leads to generation of human iTregs with superior suppressive activity *in vitro* and reduced expression of “Treg down” genes should be considered as a basis to direct future research on iTreg generation for therapeutic purposes. To this end, stabilization of iTregs with respect to Foxp3 expression, viability and *in vivo* suppressive activity needs to be achieved in the future.

Though the suppression mechanism of the human iTregs generated by the Rapa triple combination in the present study remains unclear to date, IL-2 consumption by CD25, suppression *via* CTLA-4 and IL-10 seem unlikely to confer the superior suppressive activity *in vitro* since these molecules were expressed at comparatively low levels. Least expression of IFN-γ and *SATB1* compared to other iTregs might add to the better *in vitro* suppressive capacity of Rapa triple combination iTregs. Considering the importance of TCR signal strength on Treg induction as discussed above, the effect of Rapa on counteracting strong TCR stimulation through inhibition of the Akt/mTOR pathway is likely to play an important role here as well. Consistent with lower total cell numbers generated, one might also speculate that adding Rapa to the iTreg cultures could prevent the outgrowth of contaminating non-Tregs, thus leaving a population of most suppressive “real” Tregs.

An immanent concern of iTreg generation remains their stability, and in the future, efforts should be made to combine Treg-inducing protocols such as developed in this study with protocols both to stabilize and to assess the stability of the generated iTregs. To date, the most reliable marker to determine Treg (Foxp3) stability is TSDR demethylation. Most studies find that iTregs display only intermediate TSDR demethylation if at all, concomitant with intermediate suppressive function not comparable to nTregs. Since we tested several new protocols here, as well as used higher TGF-β concentrations to induce human iTregs compared to others [58], we evaluated TSDR demethylation in iTregs, but could not detect it in any of the iTreg populations generated by all different protocols. Our results are in line with reports on murine TGF-β and TGF-β + ATRA induced iTregs [5,24,26,60,61] as well as human TGF-β induced iTregs [58,106], and also TGF-β + Rapa combinations were reported to fail in inducing TSDR demethylation in human iTregs [56]. Yet, mouse iTregs generated with TGF-β plus either ATRA or Rapa or both seemed to display somewhat enhanced stability compared to TGF-β only [79], and stimulation by bead-bound CD3/CD28 was important to generate stable murine iTregs when compared to plate-bound stimulation despite unchanged TSDR methylation [63]. TSDR demethylation has not been analyzed for neither murine nor human iTregs generated with the TGF-β + ATRA + Rapa triple combination. Even though we could not observe TSDR demethylation even in Rapa triple combination iTregs, it is noteworthy that others have reported suppressive and stable murine iTregs by mTOR inhibition through progesterone without TSDR demethylation [61]. In contrast to a recent study proposing some TSDR demethylation in human iTregs after STAT3 inhibitor treatment [73], we did not obtain such results, which may be due to differences in stimulation conditions or experimental readout. The analysis of TSDR demethylation in iTregs generated with SCFAs such as butyrate has not been published before, and we now show that butyrate addition does not lead to TSDR demethylation in human iTregs. It was proposed that Foxp3 expression in iTregs is unstable because of absent TSDR demethylation [27]. Nevertheless, some recent studies find suppressive and importantly stable human and murine iTregs without strong TSDR demethylation [56,61–63], which may suggest that factors other than TSDR demethylation also contribute to Foxp3 stability. For example, it may be associated with induction of other epigenetic changes such as histone modifications as described for ATRA [107]. One may also speculate that iTregs and pTregs have distinct properties from tTregs and may not require TSDR demethylation; potentially instability might be even an intrinsic property of pTregs compared to tTregs, whether for a physiological reason remains unknown. Along these lines, it was found that Nrp1 negative pTregs isolated from healthy wild type mice were less stable compared to tTregs upon transfer into lymphopenic mice [34], which is in concordance with observed lesser TSDR demethylation in Nrp1 negative pTregs compared to Nrp1+ tTregs from wild type mice *ex vivo* [35]. Also, another report suggests that Tregs which are unstable *in vivo* may arise rather from pTregs than from tTregs [108], suggesting a pTreg subset distinct from tTregs. Of note, TSDR demethylation in tTregs is established already in the thymus [109]. Alternatively, it is possible that, even though TSDR demethylation in iTregs is not observed, it may be established upon transfer of the cells into an *in vivo* milieu. Along that note, it was described that retention of Foxp3 in iTregs may require a pro-inflammatory cytokine environment [55]. Notably, Schmitt *et al*. [110] have demonstrated that iTregs generated *in vitro* can obtain at least partial TSDR demethylation upon their maintenance *in vivo*. Similarly, Sela *et al*. observed TSDR demethylation of iTregs resembling tTregs after transfer of the iTregs into mice [111]. In line with our results on human cells, it was shown for murine iTregs that IL-2 could stabilize Foxp3 expression *in vitro*, while restimulation led to loss of Foxp3 expression [112]. It could be confirmed *in vivo* that IL-2/anti-IL-2 complexes stabilized Foxp3 expression of iTregs [112]. Strikingly, adoptively transferred *in vitro* generated murine iTregs acquired TSDR demethylation *in vivo* upon treatment of the mice with IL-2 when administered in the presence of TCR stimulation [112]. Along these lines, another study found that concurrent administration of Rapa with or without IL-2/anti-IL-2 antibody complexes to mice receiving murine iTregs improved Foxp3 stability in those transferred cells [96]. An additional effect of IL-2 supplementation might be enhancement of iTreg survival *in vivo*. Notably, IL-2 administration has been applied in human diseases and was found to increase Treg expansion *in vivo* in patients [113–115].

These results allow to speculate that also human iTregs, generated by optimized protocols considering aspects such as TCR stimulation strength, addition of Treg-inducing compounds (such as TGF-β /ATRA/Rapa triple combination as described here) and *in vivo* stabilization such as by IL-2 complexes, may be useful therapeutically—given that appropriate conditions to induce human iTreg stability through TSDR demethylation or other means *in vitro* or *in vivo* can be identified in the future.

## Materials and Methods

### Ethics statement

Human peripheral blood mononuclear cells (PBMCs) were freshly isolated from anonymized healthy donor buffy coats which were purchased from the Karolinska University Hospital (Karolinska Universitetssjukhuset, Huddinge), Sweden. Ethical permit for the experiments with human blood was obtained from the Regional Ethical Review Board in Stockholm (Regionala etikprövningsnämnden i Stockholm), Sweden (approval number: 2013/1458-31/1). Mouse experiments were performed in accordance with the national guidelines and approved by the Regional Ethical Review Board (Stockholms Norra Djurförsöksetiska nämnd) in Stockholm, Sweden (Jordbruksverket approval number N274/14). All efforts were made to minimize suffering of the mice.

### Human T cell isolations

PBMCs were purified from fresh buffy coats by gradient centrifugation using Ficoll-Paque Plus (GE Healthcare), followed by plastic adherence in RPMI medium containing 10% FCS (Invitrogen) to deplete monocytes. PBMCs were rested overnight, and different T cell subsets were then isolated by magnetic activated cell sorting (MACS). CD25high “nTregs” were first prepared by positive isolation as we described previously [116] using limited amounts (2 μl/10^7^ cells) of CD25 beads (Miltenyi) and two subsequent MACS columns. After platelet depletion by low-speed centrifugation (3x200 g, 5 min, 20°C), naïve T cells were isolated by negative isolation from the nTreg-depleted fraction using the naive CD4+ T Cell Isolation Kit II, human (Miltenyi) according to the manufacturer’s instructions. Pan T cells used as Tresp in suppression assays were isolated by negative selection with the human pan T cell isolation kit II (Miltenyi) and additionally depleted of CD25+ cells (with 8 μl CD25 beads/10^7^ cells). For APC purification, PBMCs before monocyte adherence were used and depleted of CD3+ cells with magnetic CD3 beads (Miltenyi) and subsequently γ-irradiated with 30 Gray. Cell purity of all MACS-isolated cells was assessed by FACS staining. Cells were counted in trypanblue solution with the Countess Automated Cell Counter (Life Technologies) and viability was determined by trypanblue stain and/or flow cytometry (see below). T cells were cultured at 5% CO_2_/37°C in serum-free X-Vivo 15 medium (Lonza) supplemented with 1% Glutamax (Invitrogen). iTreg and nTreg cultures were supplemented with 100 U/ml IL-2, unless stated otherwise.

### iTreg differentiation

After MACS isolation, naive T cells were rested for 3 to 8 hours and then plated under iTreg differentiation conditions at 1.1 to 1.3x10^5^ cells/well in 96U well plates. Cells were stimulated with 5 μg/ml plate-bound anti-CD3 antibody (clone OKT3; Biolegend, LEAF grade), 1 μg/ml soluble anti-CD28 antibody (Biolegend, LEAF grade) and 100 IU/ml IL-2 (carrier-free; R&D Systems). Cells stimulated with only these reagents served as “mock” control. For Treg-inducing conditions, TGF-β1 (5 ng/ml carrier-free; R&D Systems), ATRA (10 nM; Sigma-Aldrich), Rapa (100 ng/ml; Calbiochem EMD Millipore), sodium butyrate (100 μM; Sigma-Aldrich), or STAT3 inhibitor S3I-201 (50 μM; Sigma-Aldrich) were added additionally. The DMSO control (for ATRA, Rapa, STAT3i) had no effect on Foxp3 expression. Cells were incubated for 6 days unless otherwise stated.

### *In vitro* suppression assays

Before use in suppression assays, iTregs were washed on day 6 of induction and rested for 2 days without stimulation in X-Vivo 15 medium supplemented with 50 U/ml IL-2. Before setup of suppression assays, iTregs were washed again, taken up in fresh X-Vivo 15 medium without IL-2 and cocultured with Tresp as described below. Mock control suppressor cells from the same donor were generated in parallel to iTregs by stimulation plus IL-2 only and then washed and rested in the same way as iTregs. nTregs from the same donor were cryopreserved after isolation in 90% FCS/10% DMSO (Hybri-Max grade, Sigma) and stored in liquid nitrogen. Prior to use, nTregs were thawed, washed and rested 2 days in X-Vivo 15 medium containing IL-2. Tresp (CD25 depleted pan T cells) were used either fresh or cryopreserved and rested one day after isolation or thawing. Tresp were then washed with PBS and labeled with 2.5 μM CFSE (Molecular probes) and staining stopped with PBS/human serum before washing and taking up the cells in X-Vivo 15 medium. Labeled Tresp were rested overnight, then taken up in fresh medium and used for setup of suppression assays. For the standard setup assessing suppression in an APC-free system, Tresp and allogeneic suppressor cells were set up in different ratios, with constant 1x10^5^ Tresp/well in 96U well plates in X-Vivo 15 medium. 2x Tresp (2x10^5^ cells), unstimulated Tresp, or stimulated suppressor cells alone (1–2x10^5^ cells) were used as controls. Cells were stimulated with plate-bound anti-CD3 (5 μg/ml) and soluble anti-CD28 (1 μg/ml) antibodies for 3 to 5 days. Suppression was analyzed by flow cytometry (CFSE-based proliferation and intracellular IFN-γ production of CD4+ or CD8+ Tresp, combined with fixable viability dye and Foxp3 staining) after 4 hours restimulation with PMA/ionomycin. For suppression assays with APCs, T cell-depleted, irradiated PBMCs were used as APCs and plated with 1.5x10^5^ cells/well in 96F well plates. 1x Tresp (allogeneic) were added with 3x10^4^ cells/well, and suppressor cells (third party) added in different ratios. 0.25 μg/ml soluble anti-CD3 antibody was added in addition, except for unstimulated controls. Cells were cultured for 3 to 4 days and suppression was analyzed by flow cytometry as above, with additional gating on CD3+ cells.

### *In vivo* suppression assays (xenogeneic GvHD)

Female NOG mice (NOD.Cg-*Prkdc*^*scid*^ *Il2rg*^*tm1Sug*^/JicTac) were purchased from Taconic Biosciences Inc. and housed under sterile conditions in individually ventilated cages and given *ad libitum* access to autoclaved food and water in the institutional animal facility (maintained on 12 hours light/dark cycle). Mice were acclimatized for 1 to 4 weeks and experiments started at 9–10 weeks of age. For induction of xenogeneic GvHD, mice were irradiated with 2 Gy and injected intravenously with 20x10^6^ PBMCs +/- 5 x10^6^ Tregs in 200 μl PBS on the same day. As control, PBS alone was injected into irradiated mice. Prior to injection into mice, iTregs were induced for 6 days and rested for 2 days as described above. nTregs were isolated as described above from the same donor as iTregs and expanded for 1 week with anti-CD3/CD28 beads (Invitrogen; 1:1 bead:cell ratio) in the presence of 300 U/ml IL-2 and 100 ng/ml Rapa. Then, stimulation beads were removed and nTregs were washed and rested in medium containing 50 U/ml IL-2 for 2 days. Allogeneic PBMCs were freshly isolated the day before injection as described above but without monocyte adherence, and CD25+ cells were depleted by MACS separation. All cells were washed thrice in PBS, and PBMCs were combined with T cells before intravenous injection into mice. Mice were monitored for weight loss every 1 to 3 days and sacrificed when 20% weight loss was reached or when severe GvHD symptoms (hunched back, impaired movement/activity, ruffled fur) became evident.

### RNA preparation and quantitative RT-PCR (qRT-PCR)

Total RNA was isolated using the RNAqueous Micro Kit (Ambion), quantified with a Nanodrop 2000 (Thermo Scientific) and cDNA was prepared using the SuperScript^®^ VILO cDNA Synthesis Kit (Invitrogen) according to the manufacturer’s instructions. RNA from unstimulated nTregs as well as unstimulated Tnaive was sampled on day 0 to ensure high cell viability. mRNA was quantified using Taqman probes (Applied Biosystems best coverage probes, FAM reporter) with the Taqman gene expression mastermix (Applied Biosystems) and measured on a StepOne plus detector system (Applied Biosystems). The relative mRNA expression was determined by normalization to *RPL13A*. Results are presented as fold induction compared to mRNA amounts of unstimulated T naïve of the same donor, which were set to 1. Fold expression was calculated using the ΔΔCt method according to the following formula (Ct is the threshold cycle value):

$$Relative\;\;mRNA\;expression=2^{-\left(Ct\;of\;gene\;of\;interest-Ct\;of\;RPL13A\right)}$$

### Analysis of TSDR methylation

Genomic DNA of iTregs or control cells generated from male donor cells was isolated with the DNeasy Blood & Tissue Kit (Qiagen) according to the manufacturer’s instructions. Unstimulated Tnaive and nTregs were sampled on day 0 as controls. TSDR methylation analysis by Bisulfite sequencing was done by Epiontis GmbH, Berlin, Germany as previously described [58].

### Flow cytometry and antibodies

#### Surface stainings

If not otherwise stated, cell surface antigens were stained in the dark in antibody dilutions in FACS buffer (PBS/0.5% HSA) for 15 minutes at 20°C or 30 minutes at 4°C. Cells were washed once with PBS, taken up and measured in FACS buffer or used for subsequent intracellular stainings.

#### Intracellular and viability stainings

After surface staining, cells were washed twice with PBS and stained with the Fixable Viability Dye (ebioscience) for 30 minutes at 4°C (dark), then washed twice and used for intracellular stainings. Intracellular stainings were performed with the Foxp3 Staining Buffer Set (ebioscience) according to the manufacturer’s protocol at 4°C. To measure intracellular cytokines, cells were pulsed 4 h before staining with 0.5 μM ionomycin and 10 ng/ml PMA (Sigma-Aldrich) in the presence of Golgi plug (BD Biosciences). Isotype control antibodies were used in the same final concentrations (w/v) as the corresponding intracellular staining antibodies.

#### The following dyes and antibodies were used (all against the human proteins)

Foxp3-APC (ebioscience, clone 236A/E7) and mIgG1 κ APC isotype control (ebioscience, clone P3.6.2.8.1), CD25-PE (Miltenyi), CTLA-4-BV421 (BD Biosciences, clone BNI3) and mIgG2a κ BV421 isotype control (BD Biosciences), IFN-γ-PE and mIgG1 κ PE isotype control (ebioscience), IFN-γ-FITC and mIgG1 κ FITC isotype control (ebioscience), IL-17A eFluor450 and mIgG1 κ eFlour450 isotype control (ebioscience), CD45RA-PE-Vio770 (Miltenyi), CD45RA-FITC (Miltenyi), CD45RO-PE (BD Biosciences), CD4-PerCP (BD Biosciences), CD3-PE-Vio770 (Miltenyi), CD8-eFlour450 (ebioscience), fixable viability dye-eFlour780 (ebioscience), CFSE (Molecular Probes).

#### Acquisition and analysis

Acquisition was performed on a CyAn^™^ ADP 9 Color Analyzer (Beckman Coulter), and parameter compensation was performed automatically with the CyAn software (Summit) tool using single stained samples containing positive cells. FACS data were analyzed using FlowJo (Tree Star).

#### Statistical analysis

Statistical analysis was performed with GraphPad and p values <0.05 were considered significant. Bar and line charts were generated in Microsoft Office Excel, boxplots were created in R, survival curves were created and analyzed [Log-rank (Mantel Cox) test] in GraphPad Prism 6. Phenotypic differences between iTreg conditions were calculated by paired t test, comparing the different conditions for paired (within one donor) samples. *In vitro* suppression assays were analyzed with paired t test compared to mock suppressor cells, as well as with one-sample t test, as indicated.

## Supporting Information

S1 Fig Cell purity and experimental setup for iTreg differentiation. (A) Naive human CD4 T cells were isolated by the Naive CD4+ T Cell Isolation Kit II, human (Miltenyi). Naive CD4 T cell purity, based on CD4, CD45RA and CD45RO, was 94–98% and purity of a representative donor of more than 20 is shown. PBMCs from the same donor, before MACS isolation, are shown as a control. (B) *Ex vivo* Tregs (”nTregs”) were isolated by using limited amounts of CD25 microbeads (Miltenyi) and used as a positive control for iTreg experiments. Naive CD4 T cells were isolated as described in (A). Representative nTreg and Tnaive purity based on CD4 and CD25 is shown here for one donor out of more than 20. For Foxp3 expression, see Fig 1. The lower panels show CD45RA and CD25 expression in nTreg preparations for the same donor; naive T cells are shown as a comparison. (C) Experimental setup for iTreg induction and analysis. Human naive CD4 T cells were isolated from buffy coats and stimulated for 6 days in different Treg-inducing conditions (”iTreg“) or control stimulated (mock suppressor cells). Phenotypic analysis was done by flow cytometry, qRT-PCR and TSDR methylation analysis. Before use in suppression assays, iTregs were washed and rested 2 days in low IL-2, and then washed again before setup of suppression assays.
(TIF)

S2 Fig Gating strategy for iTreg phenotype analysis. Arrows indicate the gating hierarchy. As examples, different samples from day 6 are shown in A–D: (A) stimulated + IL-2, (B) stimulated + IL-2 + TGF-β, (C) unstimulated, (D) isotype control antibody stainings for intracellular stainings (for Foxp3, CTLA-4 and IFN-γ antibodies; example shown: stimulated + IL-2 + TGF-β + ATRA).
(TIF)

S3 Fig Foxp3 expression in human iTregs using different Treg-inducing conditions, kinetics and stimulation strengths. (A) Foxp3 protein expression at day 6, shown as individual lines for individual donors (each line represents one donor; except red line = mean of all donors), gated on live CD4+ cells. iTreg or control conditions are indicated on the x axis. (B) *FOXP3* mRNA expression in naive T cells cultured for 6 days under the indicated iTreg or control conditions. nTregs and unstimulated naive T cells were sampled on day 0. mRNA was quantified by Taqman assay and normalized to *RPL13A* expression. *FOXP3* mRNA expression in unstimulated naive T cells from the corresponding donor was set to 1, and fold change of *FOXP3* mRNA calculated (numbers in plot represent mean fold changes). Shown are mean +/- SEM values for n = 8 to 12 donors in 6 to 8 independent experiments. Significance was calculated with paired t test. *: p<0.05; **: p<0.01; ***: p<0.001; ****: p<0.0001. (C) Foxp3 protein expression kinetics during Treg induction on day 3 and day 6. The Treg induction (day 0 to day 6) was performed with different concentrations of anti-CD28 antibody and TGF-β as indicated, with constant 5 μg/ml plate-bound anti-CD3 and 100 U/ml IL-2. Our”standard”condition was 5 ng/ml TGF-β and 1 μg/ml anti-CD28. Unstimulated nTregs as well as unstimulated Tnaive, cultured without stimulation and with IL-2 only, are shown as controls in the upper left panel. Gate: Live CD4+ cells. One donor is shown, and the experiment was repeated with an independent donor showing similar results.
(TIF)

S4 Fig Expression of Treg signature genes in human iTregs. (A) *CTLA4* mRNA expression in naive T cells cultured 6 days under the indicated iTreg or control conditions. nTregs and unstimulated naive T cells were sampled on day 0. mRNA was quantified by Taqman assay, normalized to *RPL13A* expression. *CTLA4* mRNA expression in unstimulated naive T cells from the corresponding donor was set to 1, and fold change of *CTLA4* mRNA was calculated. Shown are mean +/- SEM values for n = 4 to 6 donors (n number indicated in the plot). Significance was calculated with paired t test. (B, C) *IRF4* and *LEF1* mRNA expression in naive T cells was determined as described in (A). n.s.: not significant. *: p<0.05.
(TIF)

S5 Fig Foxp3 expression during resting of iTregs. (A) Experimental setup for iTreg induction and subsequent analysis of Foxp3 stability during resting of iTregs. (B) *FOXP3* mRNA expression on day 6 (colored bars) of Treg induction under the indicated conditions, as well as on day 8 (white bars) after 2 days of resting. Resting was done after washing the cells on day 6 and resting them with 50 U/ml IL-2, without stimulation and without further compounds. Unstimulated nTregs as well as unstimulated Tnaive were sampled on day 0 and are shown as controls. *FOXP3* mRNA expression was quantified by qRT-PCR using Taqman assay, normalized to *RPL13A* expression. *FOXP3* mRNA expression in unstimulated naive T cells from the corresponding donor was set to 1, and fold change of *FOXP3* mRNA was calculated. Shown are mean +/- SEM values for n = 4 donors (except butyrate, n = 2); numbers in plot represent mean fold change. (C) Foxp3 protein expression kinetics during Treg induction on day 3 and day 6, as well as during resting from day 6 to day 8. The Treg induction (day 0 to day 6) was performed with different concentrations of anti-CD28 antibody and TGF-β as indicated in the plots, with constant 5 μg/ml plate-bound anti-CD3 and 100 U/ml IL-2. Resting was done after washing the cells by resting them with 50 U/ml IL-2, without stimulation and without further compounds. Resting was done without or with (half-filled symbols) TGF-β. Unstimulated nTregs as well as unstimulated Tnaive, cultured without stimulation and with IL-2 only, are shown as controls in the upper left panel. Foxp3 positive cells were quantified by intracellular staining, gated on live CD4+ cells. One donor is shown, and the experiment was repeated with an independent donor showing similar results.
(TIF)

S6 Fig Analysis of Foxp3 stability and TSDR demethylation in human iTregs. (A) Experimental setup for iTreg induction and resting (as in S5 Fig). Tregs were subsequently washed and further cultured for 5 days with restimulation, or without restimulation and with or without IL-2. (B) Cell viability on day 13, measured by FACS, under the indicated culture conditions as described in (A). The examples shown were induced during the initial 6 days of Treg induction with IL-2 + TGF-β + ATRA + Rapa and the outcome of viability results are representative of other initial Treg-inducing culture conditions. A representative donor of 4 is shown. (C) As described in (A), iTregs were induced for 6 days under the indicated conditions, and then rested for 2 days. Afterwards, Tregs were washed and further cultured for 5 days without restimulation and with IL-2, before DNA was extracted and TSDR methylation analyzed on day 13.
(TIF)

S7 Fig Analysis of Foxp3 re-induction and TSDR demethylation in human iTregs. (A) Experimental setup to analyze Foxp3 re-induction. iTregs were induced for 6 days under the indicated conditions, and then washed. To re-induce Foxp3, cells were cultured with the same Treg-inducing conditions as in the initial culture for further 5 to 7 days, or in medium with IL-2 only as a control. Unstimulated nTregs are shown for comparison. (B) Foxp3 re-induction was analyzed on day 13 as described in (A). Foxp3 intracellular stainings, gated on live CD4+ cells, for a representative donor of 2 is shown. (C) Corresponding TSDR methylation analysis of cells on day 11 of culture as described above. See (B) for the color key.
(TIF)

S8 Fig Experimental setup for iTreg *in vitro* suppression assays. iTregs generated as in S1 Fig, or control mock suppressor cells, were rested and washed and then used as suppressor cells towards CFSE-labeled responder T cells (Tresp, CD25-depleted CD4+CD8+ pan T cells). nTregs from the same donor (previously frozen, to avoid low viability of nTregs upon prolonged culture *in vitro*) were used as control. Suppression assays were set up in different Treg:Tresp ratios, and suppression read out after 3–5 days of stimulation by flow cytometry (CFSE-based proliferation and intracellular IFN-γ production).
(TIF)

S9 Fig *In vitro* suppression assay gating strategy and exemplary samples. (A) The gating strategy is indicated by arrows. The shown example is from a coculture (set up at 1:1 ratio 5 days before) of CFSE-labeled Tresp with non-CFSE-labeled mock suppressor cells (stimulated with anti-CD3/-CD28 and IL-2 before resting and setup of suppression assay). (B) Shows unstimulated, CFSE-labeled Tresp alone. (C) Shows stimulated, CFSE-labeled Tresp alone. (D) Shows a coculture (set up at 1:1 ratio 5 days before) of CFSE-labeled Tresp with non-CFSE-labeled”TGF-β + ATRA + Rapa”iTregs. (E) Shows a coculture (set up at 1:1 ratio 5 days before) of CFSE-labeled Tresp with non-CFSE-labeled nTregs (which were frozen and thawed before setup of suppression assay). (F) Shows stimulated iTregs alone (here: ”TGF-β + ATRA + Rapa”iTregs). (G) Shows the relevant plots for isotype control stainings for intracellular antigens (the shown example is from a coculture of CFSE-labeled Tresp with non-CFSE-labeled iTregs.) All samples were pulsed with PMA/ionomycin for 4 hours before staining (except unstimulated Tresp). In suppression assays with APCs (not shown here), it was pre-gated on live CD3+ cells in addition.
(TIF)

S10 Fig Comparative analysis of *in vitro* suppressive function of human iTregs generated with different iTreg-inducing protocols. (A) For the suppression assays from Fig 5, it was tested whether there is significant suppression (1 = 100% suppression) of responder T cell proliferation against the null hypothesis = 0 = no suppression by one-sample t test, for each iTreg or control condition. The left panel shows data for CD4+ Tresp and the right panel for CD8+ Tresp. Data are presented as mean +/- SEM of n = 4 to 6 donors, for the coculture ratios Tresp:Treg of 1:1 and 1:0.5. Significant suppression is indicated by an asterisk. *: p<0.05; **: p<0.01. (B) CFSE histogram overlays of a suppression assay stimulated with anti-CD3 and APCs for the Tresp:Treg ratio of 1:1, gated on CD4 Tresp (left) or CD8 Tresp (right), for a representative donor of two. Dotted black lines = 2:0 Tresp; solid black lines = 1:0 Tresp; filled histogram = unstimulated Tresp. Color code as in (C). Lines in same color represent cells plated in replicate wells. (C) IFN-γ suppression by iTreg or control cells was measured in CD8+ Tresp. The percentage of IFN-γ positive cells is shown for one donor in the left panel, and values represent mean +/- SD of wells plated in replicate. The right panel shows percent IFN-γ suppression calculated from IFN-γ production, with 2:0 Tresp set to 100% IFN-γ production (0% suppression). Shown is the compiled data (mean +/- SEM) for n = 4 donors (except n = 2 for 1:2 ratio and butyrate iTreg condition). Significance was calculated by paired t test, comparing suppression by iTreg populations to suppression by mock suppressor cells (stimulated with anti-CD3/-CD28 and IL-2 only; grey line) at 1:1; 1:0.5 and 1:0.25 ratio, respectively. Asterisks indicate significant differences and are depicted in the color of the respective Treg condition. *: p<0.05.
(TIF)

S1 Table Individual-level data behind means of results and figures mentioned in the text. The table shows individual data points for each donor, related to flow cytometry and Q-PCR results that are summarized in the manuscript text and figures for n donors as indicated.
(XLSX)
