# Supplementary figures and images for: Comparative Analysis of Protocols to Induce Human CD4+Foxp3+ Regulatory T Cells by Combinations of IL-2, TGF-beta, Retinoic Acid, Rapamycin and Butyrate
Source: PLoS One. 2016 Feb 17;11(2):e0148474. doi: 10.1371/journal.pone.0148474 (PMC4757416; doi:10.1371/journal.pone.0148474)

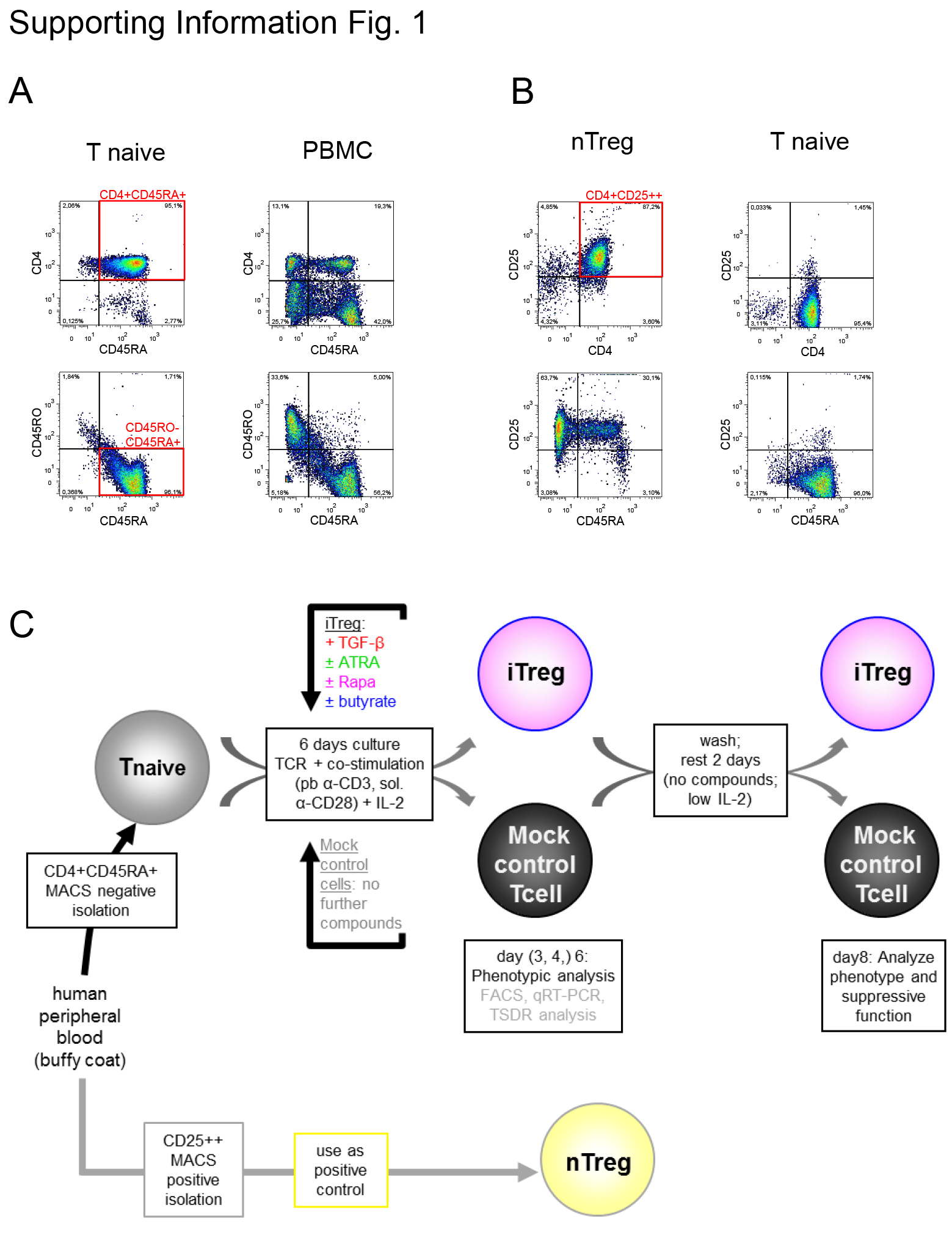

Supplement: S1 Fig — (A) Naive human CD4 T cells were isolated by the Naive CD4+ T Cell Isolation Kit II, human (Miltenyi). Naive CD4 T cell purity, based on CD4, CD45RA and CD45RO, was 94–98% and purity of a representative donor of more than 20 is shown. PBMCs from the same donor, before MACS isolation, are shown as a control. (B) Ex vivo Tregs (”nTregs”) were isolated by using limited amounts of CD25 microbeads (Miltenyi) and used as a positive control for iTreg experiments. Naive CD4 T cells were isolated as described in (A). Representative nTreg and Tnaive purity based on CD4 and CD25 is shown here for one donor out of more than 20. For Foxp3 expression, see Fig 1. The lower panels show CD45RA and CD25 expression in nTreg preparations for the same donor; naive T cells are shown as a comparison. (C) Experimental setup for iTreg induction and analysis. Human naive CD4 T cells were isolated from buffy coats and stimulated for 6 days in different Treg-inducing conditions (”iTreg“) or control stimulated (mock suppressor cells). Phenotypic analysis was done by flow cytometry, qRT-PCR and TSDR methylation analysis. Before use in suppression assays, iTregs were washed and rested 2 days in low IL-2, and then washed again before setup of suppression assays. (TIF) [file pone.0148474.s001.tif]

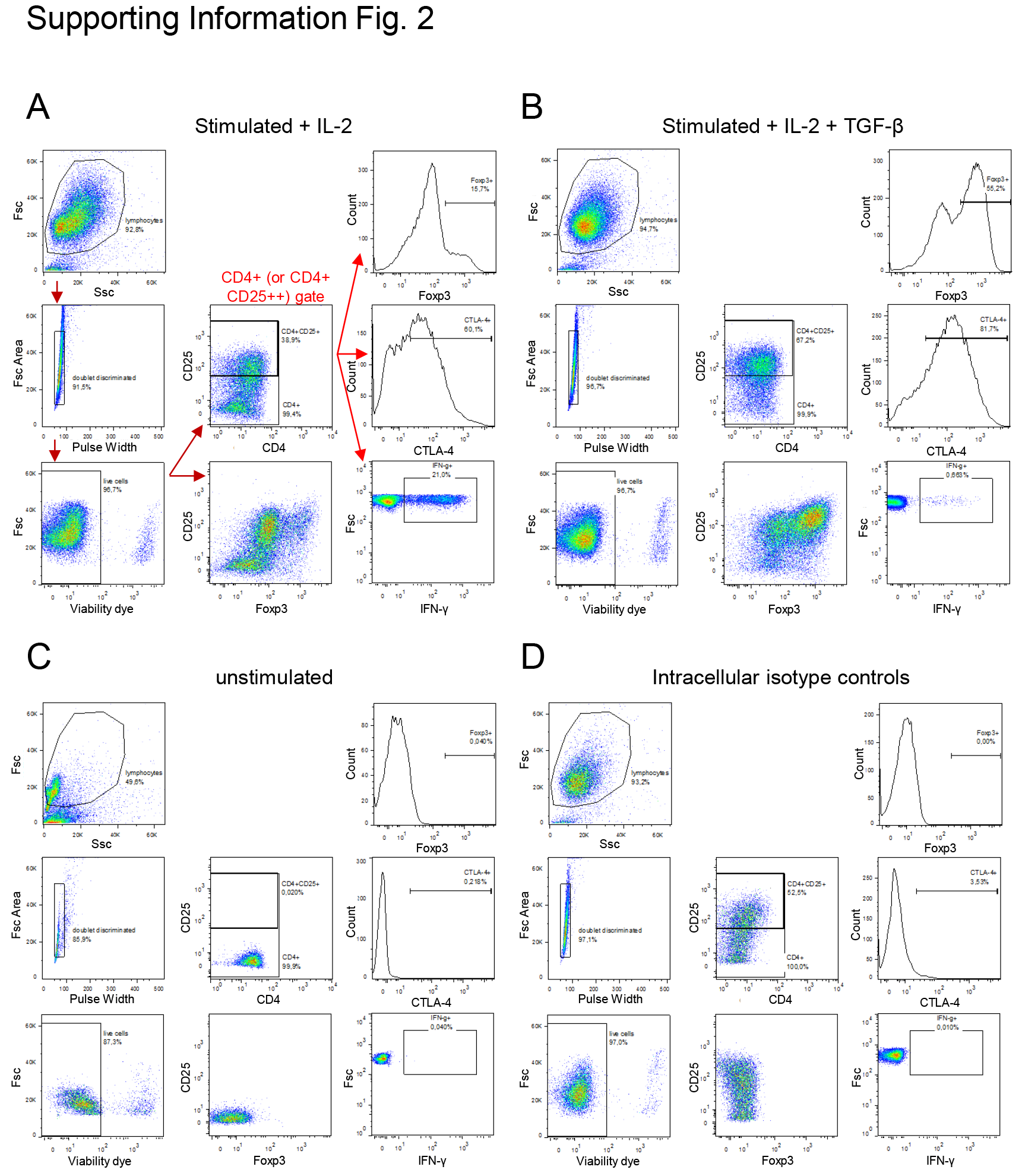

Supplement: S2 Fig — Arrows indicate the gating hierarchy. As examples, different samples from day 6 are shown in A–D: (A) stimulated + IL-2, (B) stimulated + IL-2 + TGF-β, (C) unstimulated, (D) isotype control antibody stainings for intracellular stainings (for Foxp3, CTLA-4 and IFN-γ antibodies; example shown: stimulated + IL-2 + TGF-β + ATRA). (TIF) [file pone.0148474.s002.tif]

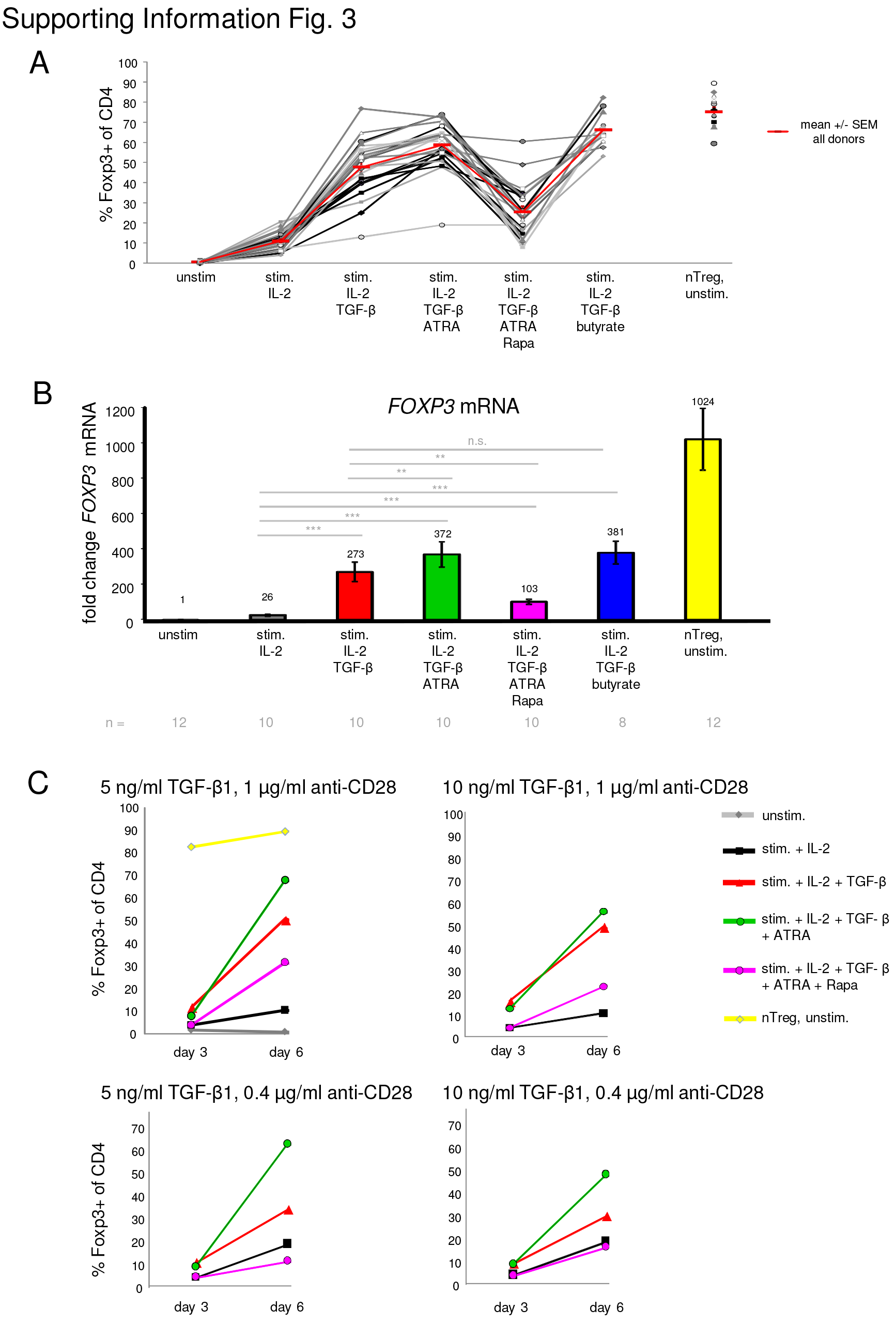

Supplement: S3 Fig — (A) Foxp3 protein expression at day 6, shown as individual lines for individual donors (each line represents one donor; except red line = mean of all donors), gated on live CD4+ cells. iTreg or control conditions are indicated on the x axis. (B) FOXP3 mRNA expression in naive T cells cultured for 6 days under the indicated iTreg or control conditions. nTregs and unstimulated naive T cells were sampled on day 0. mRNA was quantified by Taqman assay and normalized to RPL13A expression. FOXP3 mRNA expression in unstimulated naive T cells from the corresponding donor was set to 1, and fold change of FOXP3 mRNA calculated (numbers in plot represent mean fold changes). Shown are mean +/- SEM values for n = 8 to 12 donors in 6 to 8 independent experiments. Significance was calculated with paired t test. *: p<0.05; **: p<0.01; ***: p<0.001; ****: p<0.0001. (C) Foxp3 protein expression kinetics during Treg induction on day 3 and day 6. The Treg induction (day 0 to day 6) was performed with different concentrations of anti-CD28 antibody and TGF-β as indicated, with constant 5 μg/ml plate-bound anti-CD3 and 100 U/ml IL-2. Our”standard”condition was 5 ng/ml TGF-β and 1 μg/ml anti-CD28. Unstimulated nTregs as well as unstimulated Tnaive, cultured without stimulation and with IL-2 only, are shown as controls in the upper left panel. Gate: Live CD4+ cells. One donor is shown, and the experiment was repeated with an independent donor showing similar results. (TIF) [file pone.0148474.s003.tif]

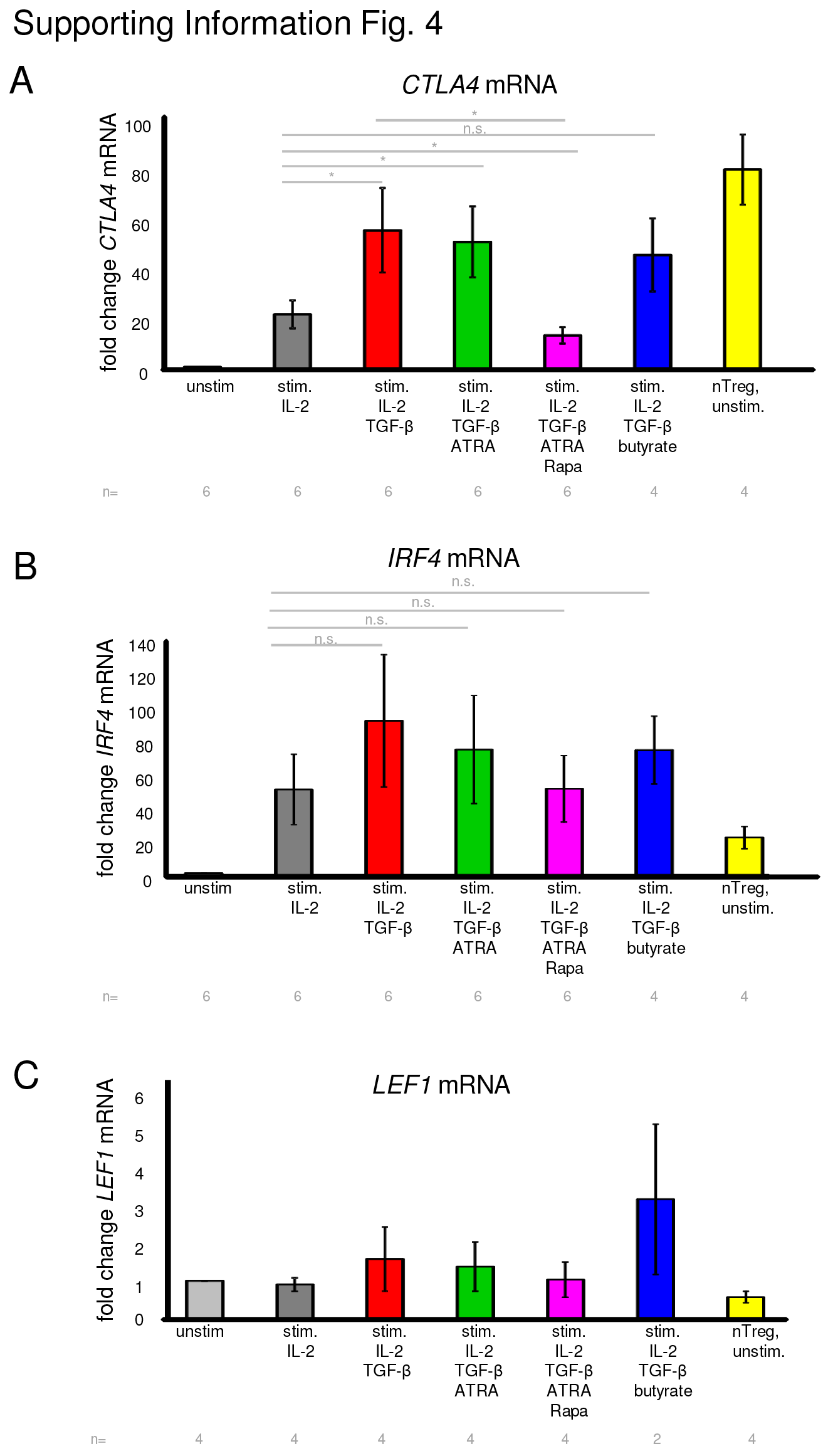

Supplement: S4 Fig — (A) CTLA4 mRNA expression in naive T cells cultured 6 days under the indicated iTreg or control conditions. nTregs and unstimulated naive T cells were sampled on day 0. mRNA was quantified by Taqman assay, normalized to RPL13A expression. CTLA4 mRNA expression in unstimulated naive T cells from the corresponding donor was set to 1, and fold change of CTLA4 mRNA was calculated. Shown are mean +/- SEM values for n = 4 to 6 donors (n number indicated in the plot). Significance was calculated with paired t test. (B, C) IRF4 and LEF1 mRNA expression in naive T cells was determined as described in (A). n.s.: not significant. *: p<0.05. (TIF) [file pone.0148474.s004.tif]

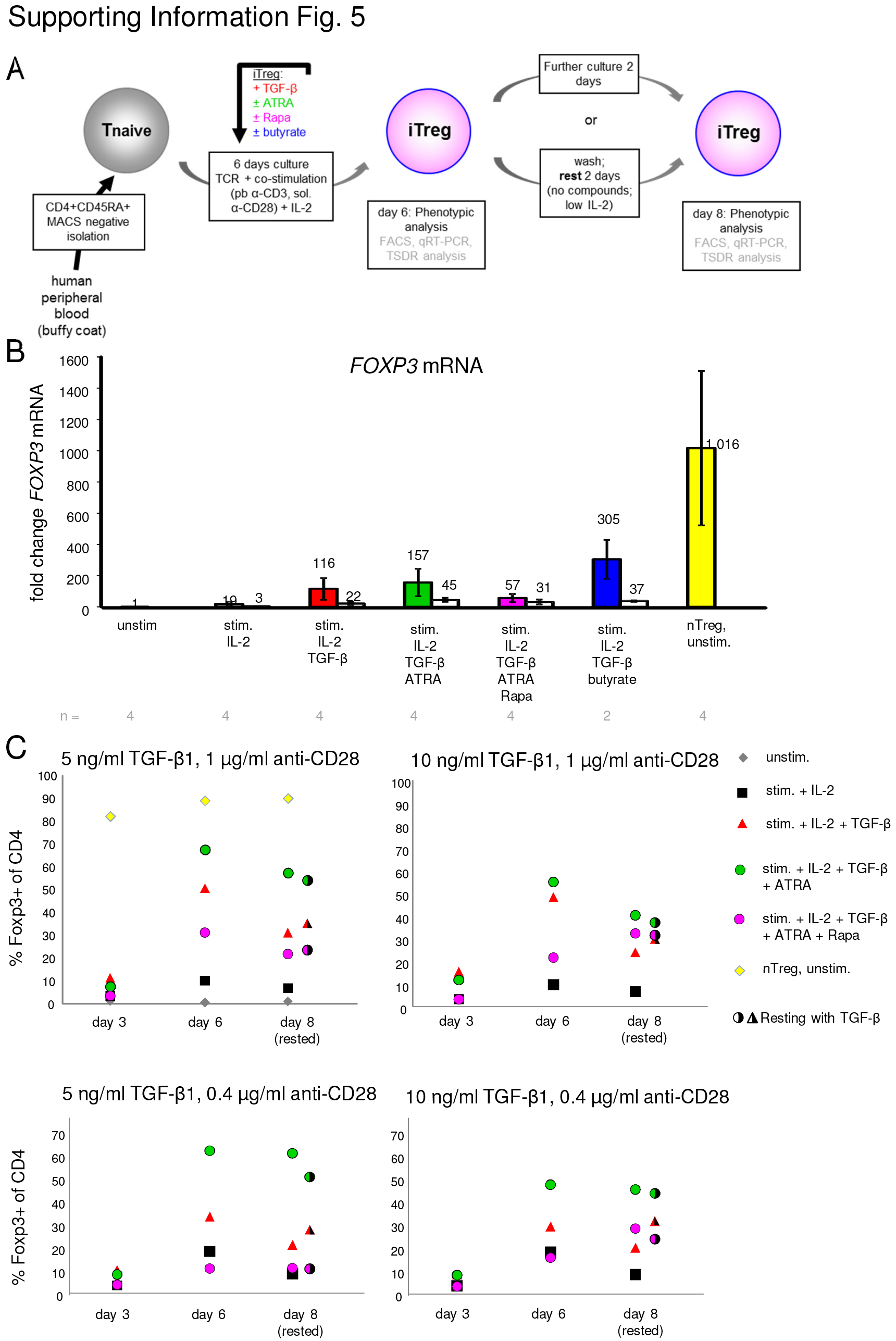

Supplement: S5 Fig — (A) Experimental setup for iTreg induction and subsequent analysis of Foxp3 stability during resting of iTregs. (B) FOXP3 mRNA expression on day 6 (colored bars) of Treg induction under the indicated conditions, as well as on day 8 (white bars) after 2 days of resting. Resting was done after washing the cells on day 6 and resting them with 50 U/ml IL-2, without stimulation and without further compounds. Unstimulated nTregs as well as unstimulated Tnaive were sampled on day 0 and are shown as controls. FOXP3 mRNA expression was quantified by qRT-PCR using Taqman assay, normalized to RPL13A expression. FOXP3 mRNA expression in unstimulated naive T cells from the corresponding donor was set to 1, and fold change of FOXP3 mRNA was calculated. Shown are mean +/- SEM values for n = 4 donors (except butyrate, n = 2); numbers in plot represent mean fold change. (C) Foxp3 protein expression kinetics during Treg induction on day 3 and day 6, as well as during resting from day 6 to day 8. The Treg induction (day 0 to day 6) was performed with different concentrations of anti-CD28 antibody and TGF-β as indicated in the plots, with constant 5 μg/ml plate-bound anti-CD3 and 100 U/ml IL-2. Resting was done after washing the cells by resting them with 50 U/ml IL-2, without stimulation and without further compounds. Resting was done without or with (half-filled symbols) TGF-β. Unstimulated nTregs as well as unstimulated Tnaive, cultured without stimulation and with IL-2 only, are shown as controls in the upper left panel. Foxp3 positive cells were quantified by intracellular staining, gated on live CD4+ cells. One donor is shown, and the experiment was repeated with an independent donor showing similar results. (TIF) [file pone.0148474.s005.tif]

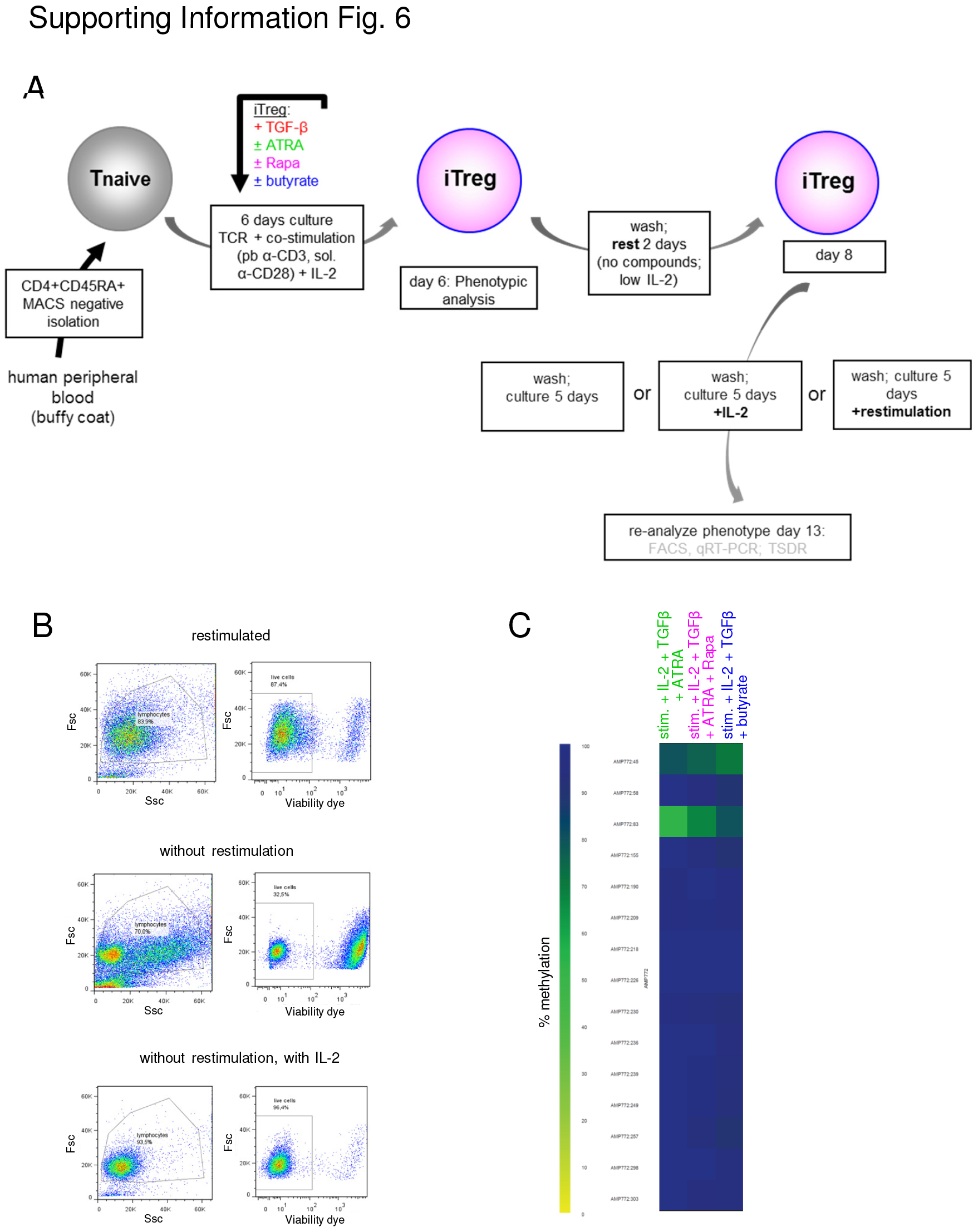

Supplement: S6 Fig — (A) Experimental setup for iTreg induction and resting (as in S5 Fig). Tregs were subsequently washed and further cultured for 5 days with restimulation, or without restimulation and with or without IL-2. (B) Cell viability on day 13, measured by FACS, under the indicated culture conditions as described in (A). The examples shown were induced during the initial 6 days of Treg induction with IL-2 + TGF-β + ATRA + Rapa and the outcome of viability results are representative of other initial Treg-inducing culture conditions. A representative donor of 4 is shown. (C) As described in (A), iTregs were induced for 6 days under the indicated conditions, and then rested for 2 days. Afterwards, Tregs were washed and further cultured for 5 days without restimulation and with IL-2, before DNA was extracted and TSDR methylation analyzed on day 13. (TIF) [file pone.0148474.s006.tif]

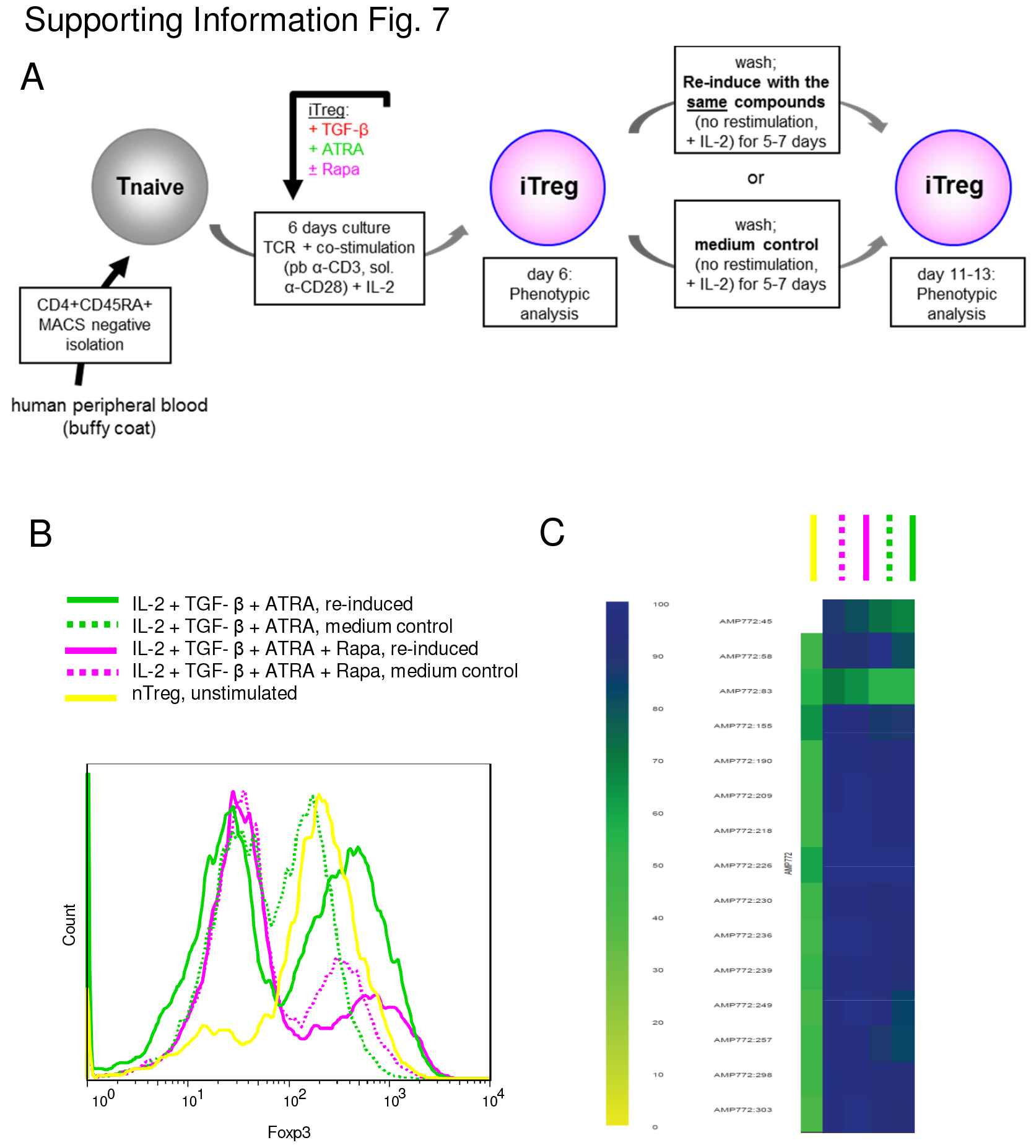

Supplement: S7 Fig — (A) Experimental setup to analyze Foxp3 re-induction. iTregs were induced for 6 days under the indicated conditions, and then washed. To re-induce Foxp3, cells were cultured with the same Treg-inducing conditions as in the initial culture for further 5 to 7 days, or in medium with IL-2 only as a control. Unstimulated nTregs are shown for comparison. (B) Foxp3 re-induction was analyzed on day 13 as described in (A). Foxp3 intracellular stainings, gated on live CD4+ cells, for a representative donor of 2 is shown. (C) Corresponding TSDR methylation analysis of cells on day 11 of culture as described above. See (B) for the color key. (TIF) [file pone.0148474.s007.tif]

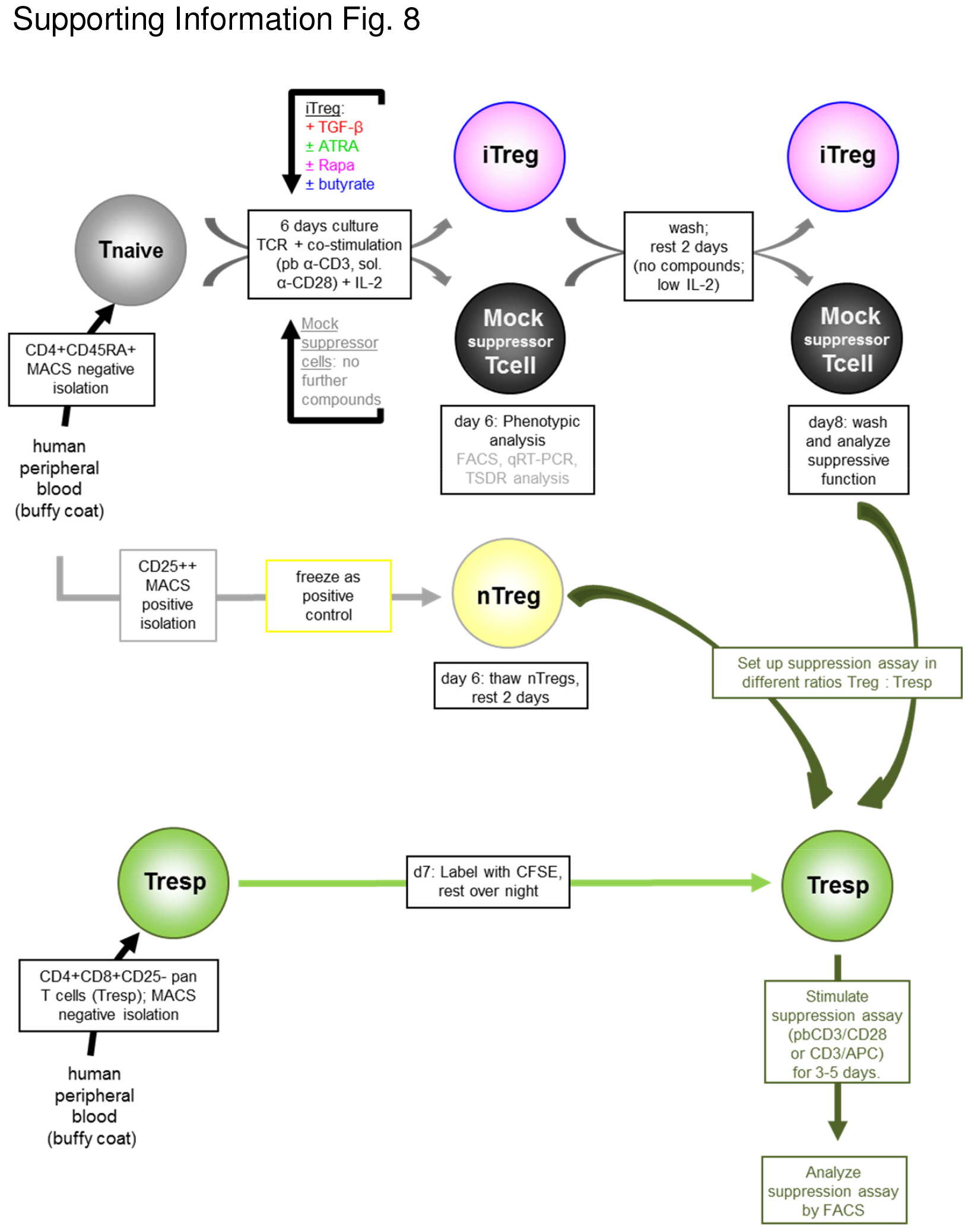

Supplement: S8 Fig — iTregs generated as in S1 Fig, or control mock suppressor cells, were rested and washed and then used as suppressor cells towards CFSE-labeled responder T cells (Tresp, CD25-depleted CD4+CD8+ pan T cells). nTregs from the same donor (previously frozen, to avoid low viability of nTregs upon prolonged culture in vitro) were used as control. Suppression assays were set up in different Treg:Tresp ratios, and suppression read out after 3–5 days of stimulation by flow cytometry (CFSE-based proliferation and intracellular IFN-γ production). (TIF) [file pone.0148474.s008.tif]

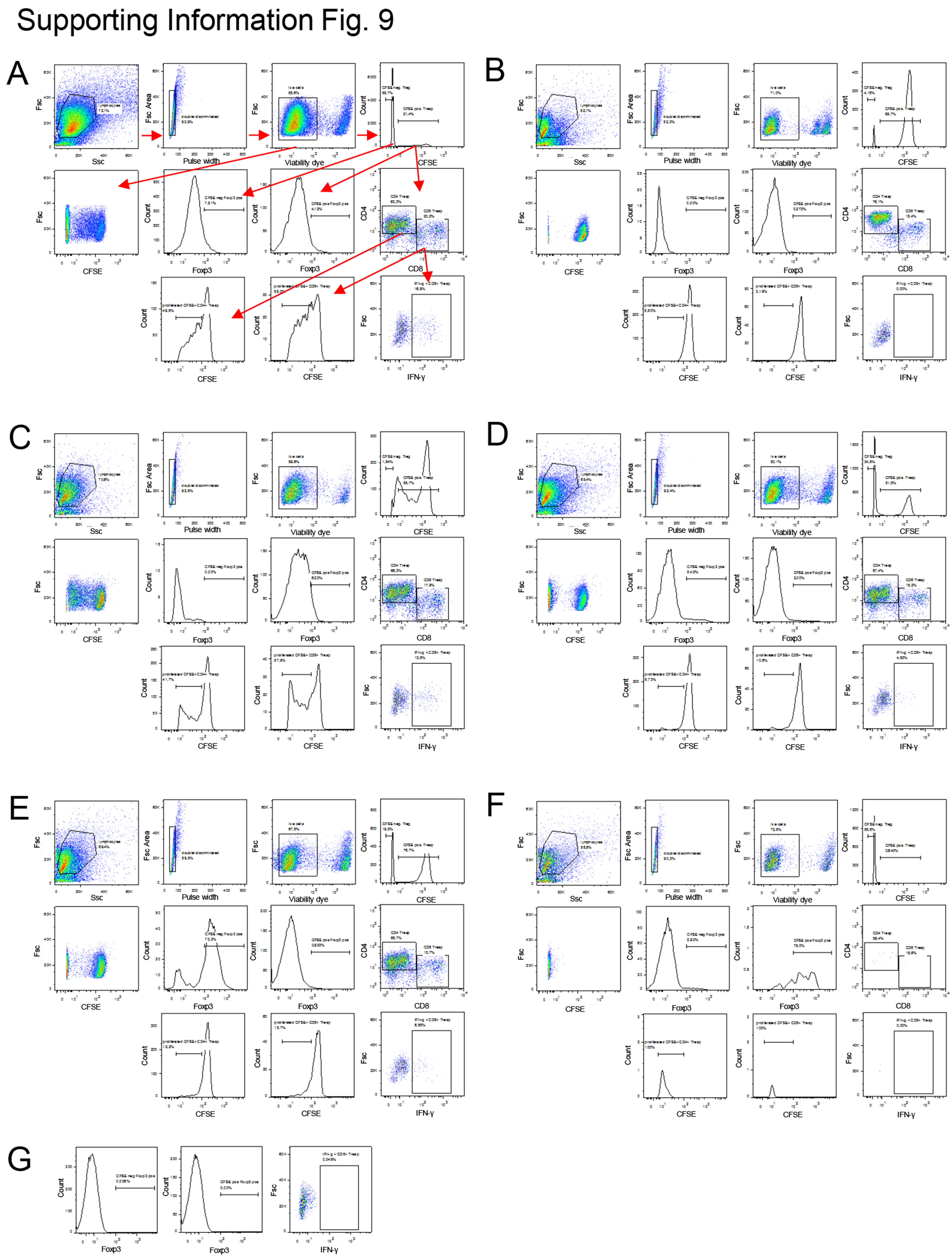

Supplement: S9 Fig — (A) The gating strategy is indicated by arrows. The shown example is from a coculture (set up at 1:1 ratio 5 days before) of CFSE-labeled Tresp with non-CFSE-labeled mock suppressor cells (stimulated with anti-CD3/-CD28 and IL-2 before resting and setup of suppression assay). (B) Shows unstimulated, CFSE-labeled Tresp alone. (C) Shows stimulated, CFSE-labeled Tresp alone. (D) Shows a coculture (set up at 1:1 ratio 5 days before) of CFSE-labeled Tresp with non-CFSE-labeled”TGF-β + ATRA + Rapa”iTregs. (E) Shows a coculture (set up at 1:1 ratio 5 days before) of CFSE-labeled Tresp with non-CFSE-labeled nTregs (which were frozen and thawed before setup of suppression assay). (F) Shows stimulated iTregs alone (here: ”TGF-β + ATRA + Rapa”iTregs). (G) Shows the relevant plots for isotype control stainings for intracellular antigens (the shown example is from a coculture of CFSE-labeled Tresp with non-CFSE-labeled iTregs.) All samples were pulsed with PMA/ionomycin for 4 hours before staining (except unstimulated Tresp). In suppression assays with APCs (not shown here), it was pre-gated on live CD3+ cells in addition. (TIF) [file pone.0148474.s009.tif]

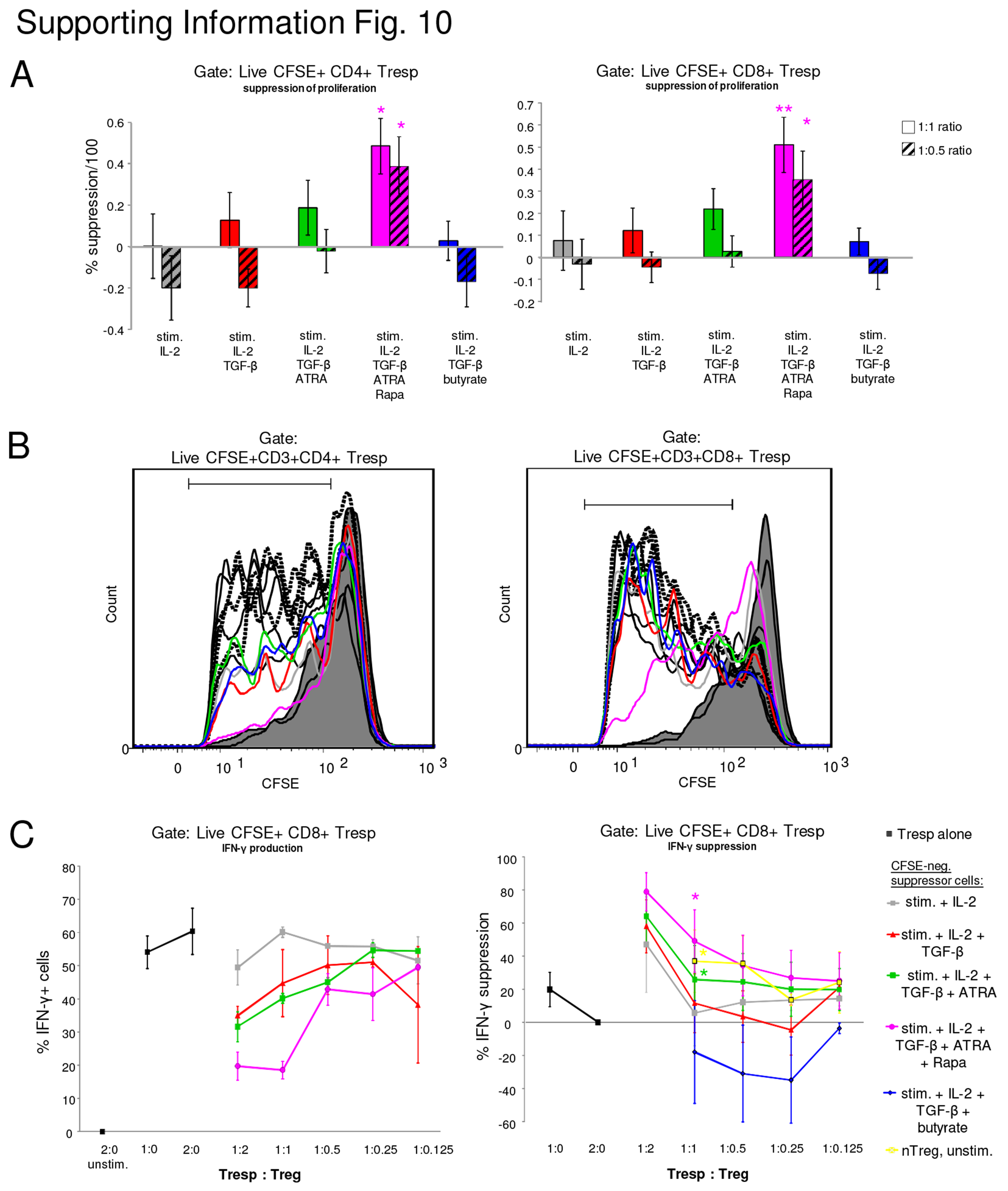

Supplement: S10 Fig — (A) For the suppression assays from Fig 5, it was tested whether there is significant suppression (1 = 100% suppression) of responder T cell proliferation against the null hypothesis = 0 = no suppression by one-sample t test, for each iTreg or control condition. The left panel shows data for CD4+ Tresp and the right panel for CD8+ Tresp. Data are presented as mean +/- SEM of n = 4 to 6 donors, for the coculture ratios Tresp:Treg of 1:1 and 1:0.5. Significant suppression is indicated by an asterisk. *: p<0.05; **: p<0.01. (B) CFSE histogram overlays of a suppression assay stimulated with anti-CD3 and APCs for the Tresp:Treg ratio of 1:1, gated on CD4 Tresp (left) or CD8 Tresp (right), for a representative donor of two. Dotted black lines = 2:0 Tresp; solid black lines = 1:0 Tresp; filled histogram = unstimulated Tresp. Color code as in (C). Lines in same color represent cells plated in replicate wells. (C) IFN-γ suppression by iTreg or control cells was measured in CD8+ Tresp. The percentage of IFN-γ positive cells is shown for one donor in the left panel, and values represent mean +/- SD of wells plated in replicate. The right panel shows percent IFN-γ suppression calculated from IFN-γ production, with 2:0 Tresp set to 100% IFN-γ production (0% suppression). Shown is the compiled data (mean +/- SEM) for n = 4 donors (except n = 2 for 1:2 ratio and butyrate iTreg condition). Significance was calculated by paired t test, comparing suppression by iTreg populations to suppression by mock suppressor cells (stimulated with anti-CD3/-CD28 and IL-2 only; grey line) at 1:1; 1:0.5 and 1:0.25 ratio, respectively. Asterisks indicate significant differences and are depicted in the color of the respective Treg condition. *: p<0.05. (TIF) [file pone.0148474.s010.tif]
